# Semi-Quantitative MALDI Measurements of Blood-Based Samples for Molecular Diagnostics

**DOI:** 10.3390/molecules27030997

**Published:** 2022-02-01

**Authors:** Matthew A. Koc, Senait Asmellash, Patrick Norman, Steven Rightmyer, Joanna Roder, Robert W. Georgantas, Heinrich Roder

**Affiliations:** Biodesix Inc., Boulder, CO 80301, USA; matthew.koc@biodesix.com (M.A.K.); senait.asmellash@biodesix.com (S.A.); patrick.norman@biodesix.com (P.N.); steven.rightmyer@biodesix.com (S.R.); joanna.roder@biodesix.com (J.R.); robert.georgantas@biodesix.com (R.W.G.III)

**Keywords:** proteomics, mass spectrometry, spectral processing, set enrichment analysis, peak detection

## Abstract

Accurate and precise measurement of the relative protein content of blood-based samples using mass spectrometry is challenging due to the large number of circulating proteins and the dynamic range of their abundances. Traditional spectral processing methods often struggle with accurately detecting overlapping peaks that are observed in these samples. In this work, we develop a novel spectral processing algorithm that effectively detects over 1650 peaks with over 3.5 orders of magnitude in intensity in the 3 to 30 kD m/z range. The algorithm utilizes a convolution of the peak shape to enhance peak detection, and accurate peak fitting to provide highly reproducible relative abundance estimates for both isolated peaks and overlapping peaks. We demonstrate a substantial increase in the reproducibility of the measurements of relative protein abundance when comparing this processing method to a traditional processing method for sample sets run on multiple matrix-assisted laser desorption/ionization-time of flight (MALDI-TOF) instruments. By utilizing protein set enrichment analysis, we find a sizable increase in the number of features associated with biological processes compared to previously reported results. The new processing method could be very beneficial when developing high-performance molecular diagnostic tests in disease indications.

## 1. Introduction

Protein abundance in blood is related to outcomes in many systemic diseases and cancer. Standard measurements of known (pre-defined) proteins via enzyme-linked immunoassays (ELISAs) used in medical diagnostics typically measure small numbers of proteins [1,2,3,4], sometimes in combination with clinical attributes [5]. Due to the complexity of pathway interactions, it is likely that a multiplexed measurement of many proteins will allow for more accurate characterization of a patient cohort in a particular disease. Indeed, such diagnostic tests have been developed based on highly sensitive high-throughput matrix-assisted laser desorption/ionization (MALDI) profiling, Deep MALDI^®^ [6] analysis, which enables the simultaneous measurement of proteins varying in abundance by four orders of magnitude. These highly multiplexed data can be combined into diagnostic tests using machine learning techniques designed to work well in the clinical setting where we generally have more attributes than samples, without over-fitting [7,8]. Multiple tests in the area of oncology were developed using this approach [9,10,11,12,13,14,15,16,17,18,19,20,21].

One challenge with using MALDI profiling is the reliable definition and characterization of many hundreds to thousands of Deep MALDI peaks with a dynamic range of peak intensity varying over four orders of magnitude and with overlapping peaks in the presence of background and noise. Reliable and reproducible peak intensity estimates are necessary as input into machine learning algorithms. Typical peak picking approaches [22,23,24,25,26] often miss many peaks. They often rely on simply finding candidate peaks, either through local intensity maxima or by finding minima in the second derivatives of the intensity, and then using intensity thresholding to select real peaks from the selection of candidate peaks. Although this method is computationally fast, it can fail to detect peaks when they overlap and may struggle to work well when there are large changes in peak intensity [27,28]. Improved peak detection algorithms using a continuous wavelet transform exhibit improved peak detection, but they often may not be accurate in the case of overlapping peaks or highly asymmetric peaks [25,26]. We propose an improved peak detection approach based on characteristics of Deep MALDI spectra. We separate well-defined (using the measured m/z, mass-charge ratio, dependent peak half-width) individual peaks from broad structures. These well-defined peaks are then fitted using a pre-defined peak shape function either individually, when isolated, or in a multi-peak fit algorithm, when overlapping. Finally, the intensity of the broad structures is added back to the intensity of the previously estimated well-defined peaks to give an expression value for a peak.

In this paper, we start by describing the data arising from a typical Deep MALDI application, and the associated peak shape function. Then, we show how to separate well-defined peaks from broad structures, and how to fit the list of peaks. We evaluate our approach on spectra from two different MALDI-TOF instruments using coefficients of variation (CVs) as the primary metric. Furthermore, we compare these results with those obtained from a common peak-fitting software package. Finally, we examine the association of the peak intensities with biological processes using set enrichment techniques [6,29] to evaluate the amount of useful biological information extracted from the spectra.

## 2. Results

Deep MALDI spectra were collected on two different MALDI-TOF instruments: the Bruker RapifleX (Bruker, Billerica, MA, USA) and the SimulTOF100 (SimulTOF Systems, Marlborough, MA, USA). Unless stated otherwise, the results shown in the main text are from the RapifleX and the corresponding results on the SimulTOF100 are shown in the Supplementary Materials.

**Figure 1:**
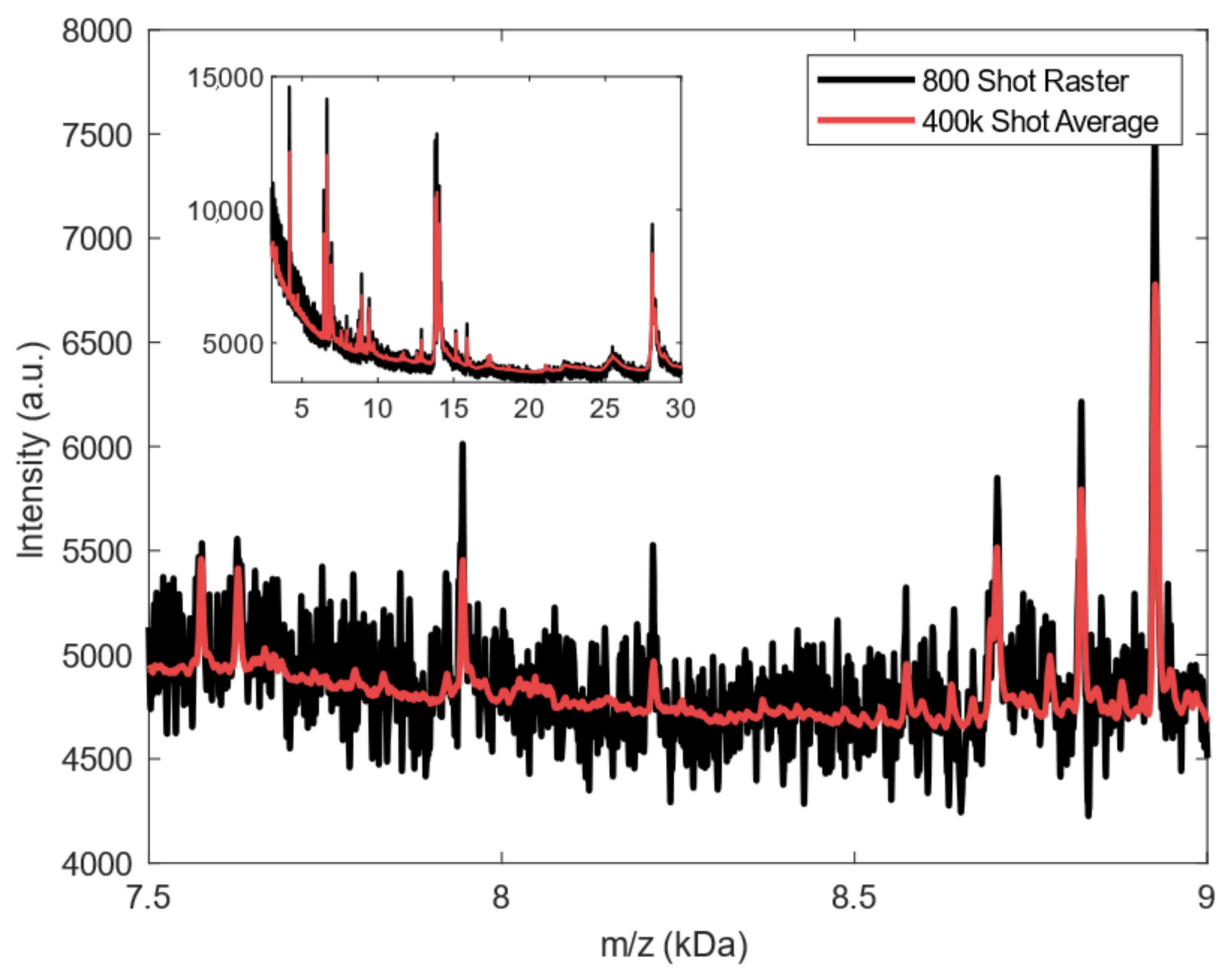
Example spectra collected on the RapifleX of an individual raster spectrum (black) and a 400,000 shot Deep MALDI averaged spectrum (red) from 7.5 to 9 kDa m/z range. The inset shows the same spectra over the full 3 to 30 kDa range analyzed in this work.

In the Deep MALDI process, for each sample preparation, multiple 800 laser shot (“raster”) spectra are collected. Individual raster spectra have significant noise, and only the strongest peaks can be accurately resolved, as shown in Figure 1. To improve the measurement sensitivity and to decrease the noise one averages 500 aligned raster spectra to create a single 400,000 shot-averaged spectrum. The 400,000 shot Deep MALDI averaged spectrum shows a greatly improved signal-to-noise ratio (SNR) and well-defined peaks are now visible that were previously hidden within the noise of a single raster spectrum. Although the sensitivity of our Deep MALDI spectra could be improved further by averaging more individual rasters, we determined that the 400,000 shot-averaged spectra result is a good compromise between sensitivity and instrument run time. We refer readers to Tsypin et al. [6] for further information on this technique.

**Figure 2:**
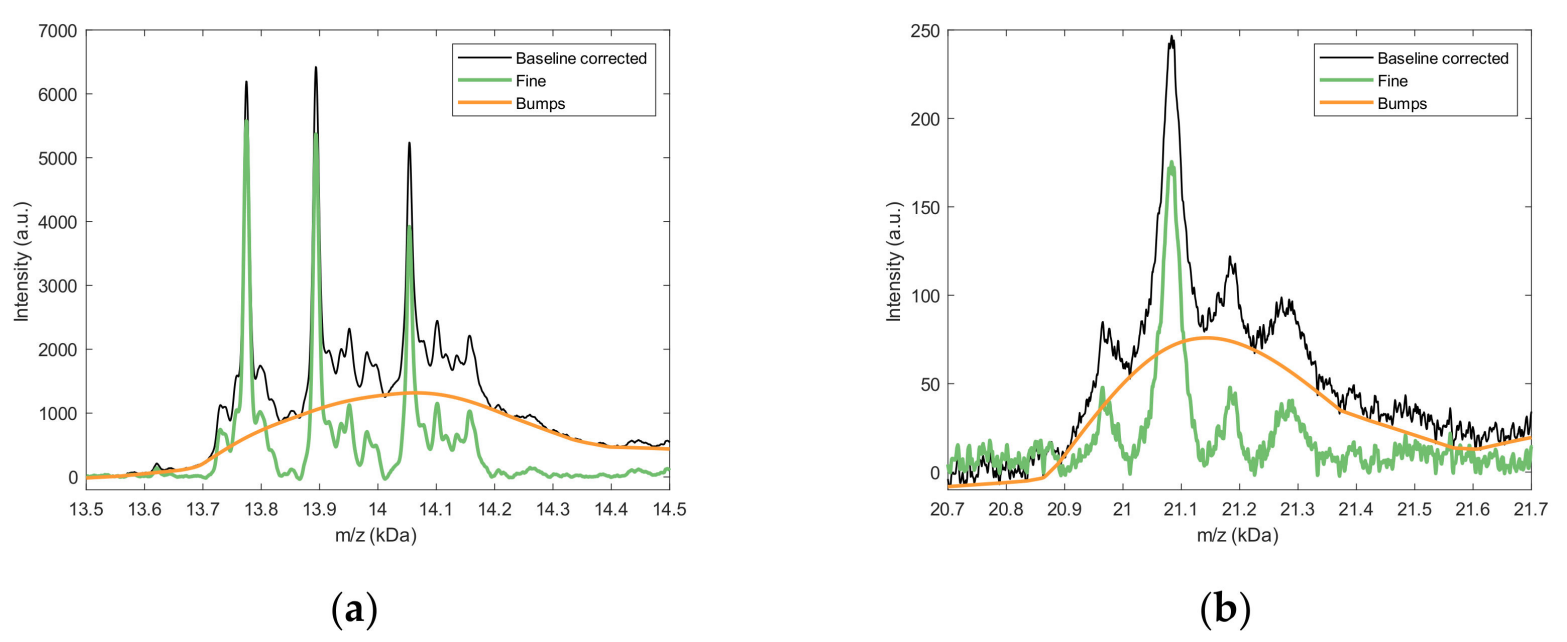
Spectral component analysis showing the baseline corrected Deep MALDI spectrum (black), fine structure (green), and bumps (orange) for peak clusters around (**a**) 14 kDa and (**b**) 21 kDa.

Because of the large number of proteins and peptides in serum samples, we observe complex structure in the baseline-corrected spectra. We often find that a baseline-corrected spectrum contains sharp features (peaks) sitting atop broad, wide features (“bumps”), as shown in Figure 2. The origin of the peaks is easy to understand as coming from proteins or peptides of a given mass. The bumps can be attributed to unresolved peaks, e.g., those arising from clusters of highly overlapping mixtures of prominent and less prominent peaks, or from multiply charged, higher-mass proteins (see Figure 2b). Because the bumps originate from biological content in the sample and are not purely an artifact of the measurement process, including the background, removing the bumps during the baselining process will reduce the potential information content available in a single spectrum. To address this problem, we separate and analyze these two components of the spectrum: the peaks (or “fine structure”) and the bumps (“bumps”). As detailed below, we find better reproducibility when we include information from both the fine structure and the bumps when determining the feature values for each peak. To maintain a consistent naming convention used in previously published literature [6,7,11,12,13,14,15], we will use the general term “feature” to refer to the peaks and “feature value” to be the semi-quantitative numerical value we calculate to represent the relative abundance of that feature (protein or peptide) within the sample. High-resolution images of a representative unprocessed and processed MALDI-TOF spectrum across the entire acquisition range (m/z = 3 to 30 kDa) is shown in the Supplementary Materials Figure S1.

### 2.1. MALDI Peak Shape Analysis

To improve upon the accuracy of our peak detection algorithm, particularly for overlapping peaks, we utilize a convolution approach whereby the spectrum is convoluted with the peak shape function of the instrument. Previous work has successfully used Gaussian functions to describe the peak shape of MALDI-TOF mass spectra [28,30], but we find this simpler approach insufficient, especially at higher masses. Individual peaks that we have observed in typical spectra are asymmetrically broadened, with the right side (high-mass side) being wider than the left side (low-mass side). This asymmetric broadening comes from a convolution of the instrument broadening and the isotope distribution, which are m/z and mass dependent, respectively. The overall peak shape of the peaks in our spectra were empirically determined to fit well to an asymmetric Gaussian of the form

(1) $$s\left(m\right)=\left\{\begin{array}{ll} A_{0}e^{-{\left(m-m_{0}\right)}^{2}ln\left(2\right)/\sigma_{L}^{2}}, & m<m_{0}\\ A_{0}e^{-{\left(m-m_{0}\right)}^{2}ln\left(2\right)/\sigma_{R}^{2}}, & m\geq m_{0}, \end{array}\right.$$

where $A_{0}$ is the amplitude, $m_{0}$ is the peak center, and $\sigma_{L}$ and $\sigma_{R}$ are the left and right half widths at half max (HWHM), respectively.

**Figure 3:**
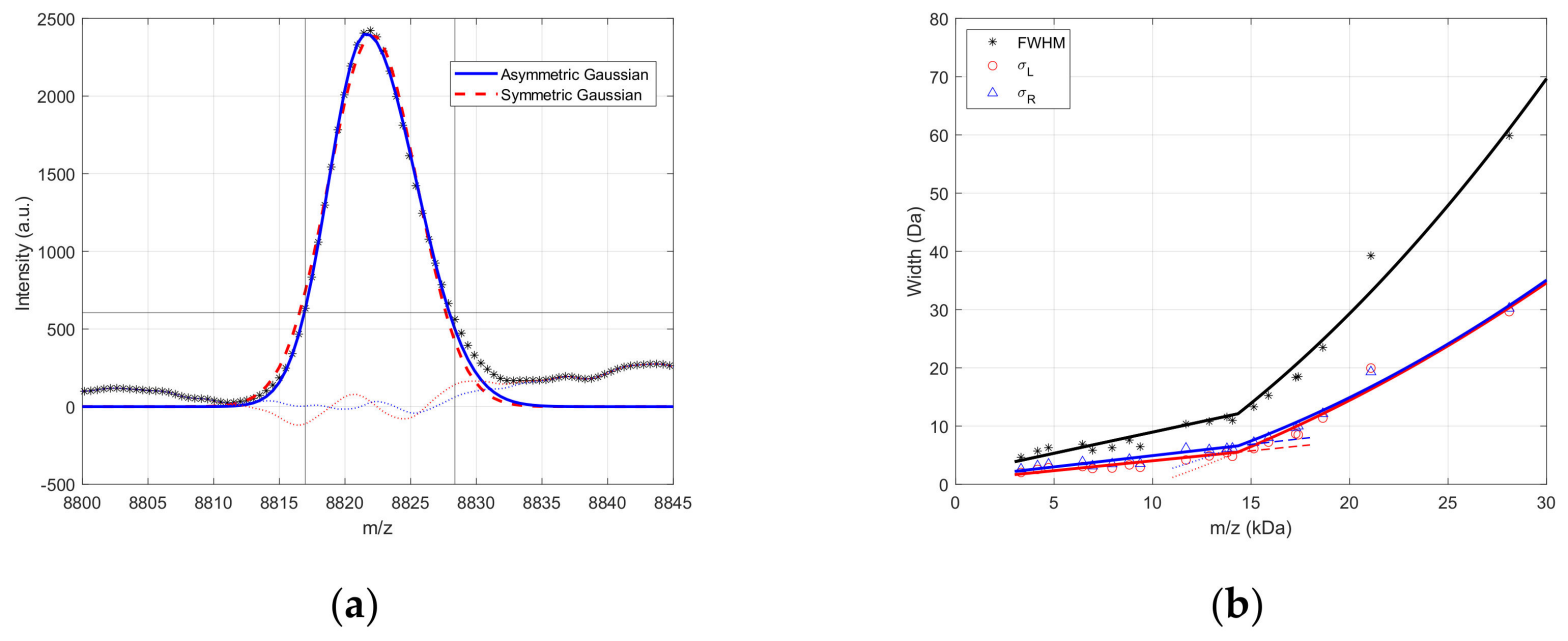
Peak shape determination of Bruker RapifleX MALDI-TOF spectral peaks. (**a**) Raw sample data (black stars) and peak fit to an asymmetric (blue-solid) and symmetric (red-dashed) Gaussian. Fit error is shown by the dotted lines. (**b**) Peak shape parameters as a function of m/z. Overall fitted trend is shown with solid lines and the linear (dashed) and quadratic (dotted) piecewise portions for $\sigma_{L}$ and $\sigma_{R}$ of the fits are extended past the trend range for reader visibility.

Figure 3a shows a typical, isolated peak in a Deep MALDI averaged spectrum and the symmetric (red-dashed) and asymmetric (blue-solid) Gaussian fits. Only data points with an intensity greater than 0.25 times the maximum intensity were used in the fit. The dotted lines show the calculated error between the raw data and the fitted peak. The sum of the absolute error in the fitting range is 1158.4 a.u. for the symmetric Gaussian fit and 150.6 a.u. for the asymmetric Gaussian fit. The asymmetric Gaussian fit shows a consistent improvement over the symmetric Gaussian fit across the entire m/z range of the peak.

A selection of 19 prominent and isolated peaks, selected to span the acquisition range, was fitted to asymmetric Gaussians to determine the m/z dependence of the peak width. Figure 3b shows the full width at half max (FWHM), $\sigma_{L}\;$, and $\sigma_{R}$ as a function of the m/z range. The right HWHM is consistently larger than the left HWHM across the range; although, at higher masses, the difference is less pronounced.

The trends of left and right HWHM across the m/z range were empirically found to fit well to a piecewise function of the m/z coordinate, *m*, as

(2) $$\sigma_{i}\left(m\right)=\left\{\begin{array}{cc} a_{0}+a_{1}m, & m<m_{int}\\ c_{0}+c_{1}m+c_{2}m^{2}, & m\geq m_{int} \end{array}\right.,$$

where $m_{int}$ is the intersection of the linear and quadratic portions of the piecewise function. The FWHM is simply

(3) $$FWHM\left(m\right)=\sigma_{L}\left(m\right)+\sigma_{R}\left(m\right).$$

The average results of 12 different reference sample preparations were used to generate the peak width parameters for the RapifleX shown in Table 1. These parameters are stable over the course of multiple months and run hundreds of samples on the instrument, as shown in the Supplementary Materials, Figure S4. The corresponding peak shape parameters for the SimulTOF100 are shown in the Supplementary Materials, Table S1, but it is worth noting that, due to the difference in instrumentation, we found that the peak shape trends fitted well to a single quadratic for the SimulTOF100 and, therefore, we set $m_{int}=0$ in Equation (1).

### 2.2. Spectral Analysis of Deep MALDI Spectra

#### 2.2.1. Background Estimation

**Figure 4:**
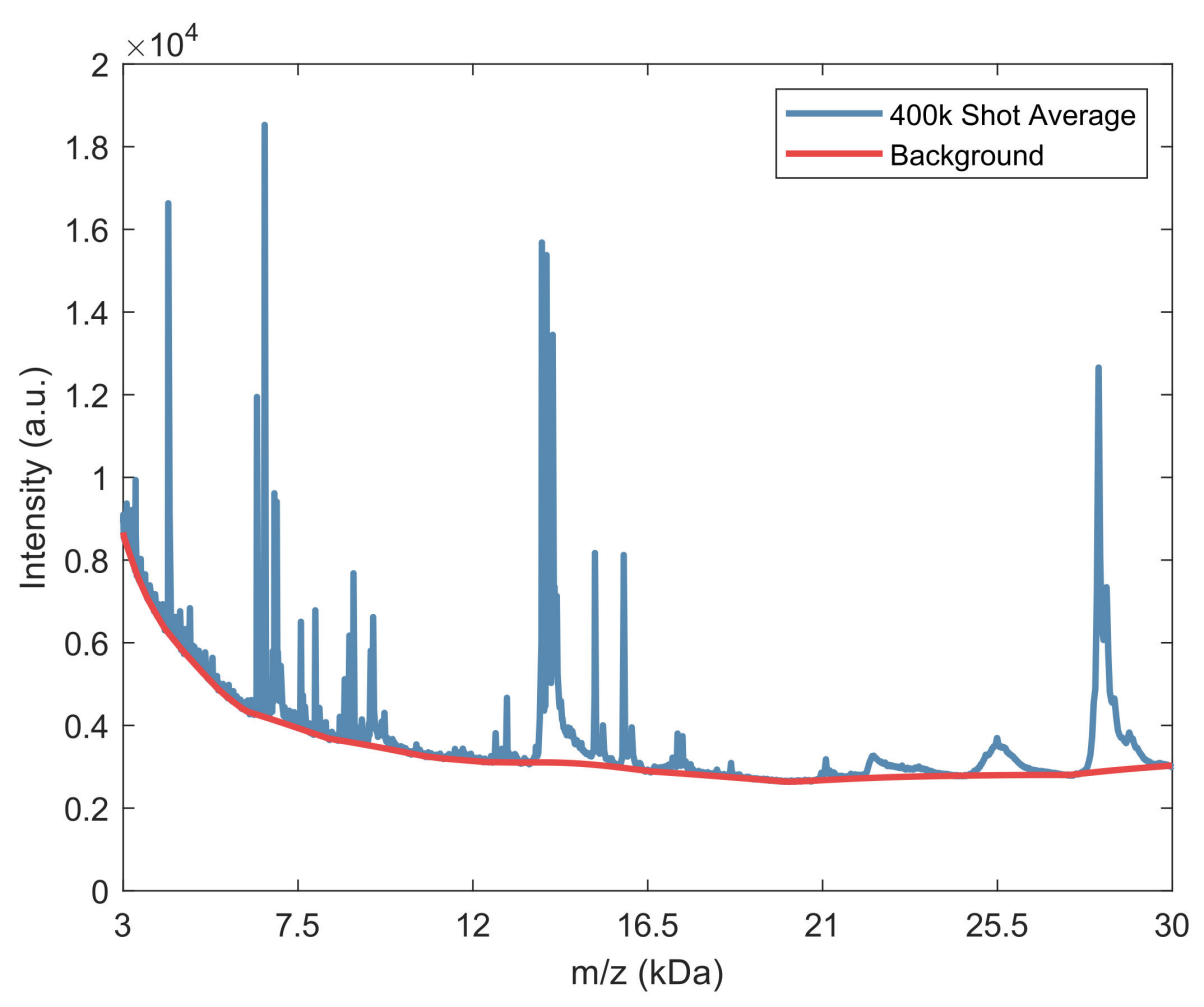
Example of a 400,000 shot-averaged Deep MALDI spectrum collected on the RapifleX and the associated Eilers’ background estimation.

The 400,000 shot average has a pronounced background that varies across the acquisition range, as shown in Figure 4. The background was estimated using Eilers’ estimation [31,32]. This method for background estimation allows for an elastic background that is penalized differently for errors above and below the background line. For our spectra, we found Eilers’ parameters of $\lambda_{1}=10^{11}$, $\lambda_{2}=10^{4}$, and p=0.001 provided a good background estimation, (*BG*_1_), without over-fitting to the spectral peaks. For further description of the Eilers’ method and selection of its parameters, we direct the reader to Boelens et al. [32].

#### 2.2.2. Fine Structure Determination and Peak Fitting

As described above, the fine structure is defined to be only the component of the MALDI spectra that contains the sharp features on a flat background. This was calculated by subtracting a relaxed Eilers’ background ($\lambda_{1}=10^{6}$, $\lambda_{2}=10^{2}$, and p=0.001) (*BG*_2_), from the Deep MALDI spectra. The bumps were calculated as the difference between the relaxed and stiff backgrounds:

(4) $$Bumps\left(m\right)=BG_{2}\left(m\right)-BG_{1}\left(m\right).$$

**Figure 5:**
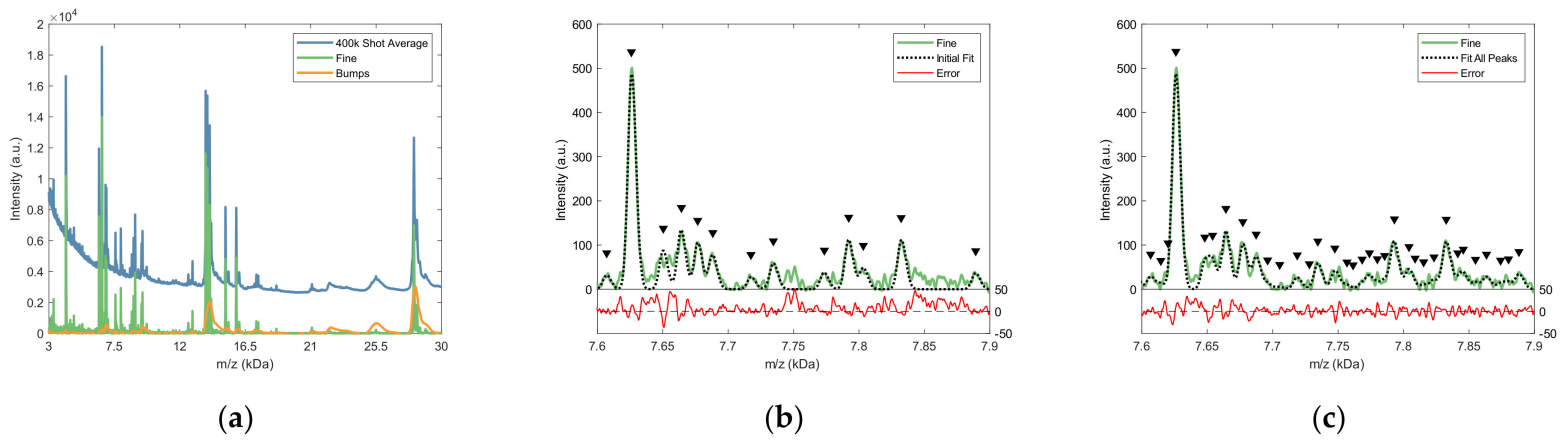
Visual representation of peak fitting and feature value determination. (**a**) A single-processed Deep MALDI spectrum showing the fine structure (green) and bumps (yellow) components. (**b**) Initial peak finding and result of applying the fitting algorithm to the fine structure of a single spectrum in the range 7.5–7.9 kDa. (**c**) The complete fitting of the same range using the master list of all peaks. The triangles indicate locations of fitted peaks and the red trace at the bottom shows the error in the peak fit.

The visual representation of the spectral components is shown in Figure 5a.

A peak-finding algorithm (see Section 4.4.4 for details) was used to determine the largest peaks that could be used to align spectra to a common m/z axis and to generate a master peak list from multiple samples. The master peak list is a collection of all unique peaks found across all samples and is used to accurately fit the entire spectrum, even peaks that are only sporadically present. Briefly, we first calculated the convolution of the spectrum fine structure with the peak shape function to differentiate true peaks from artifact structures, such as noise. The algorithm then searched for peaks that had SNR > 10 and whose centers were more than one FWHM away from adjacent peaks. Figure 5b shows a sub-range of the entire spectrum that has the fit of the peaks found by this algorithm. Typically, between 700 and 800 peaks with SNRs in this range are found for an individual 400,000 shot Deep MALDI spectrum acquired from 3–30 kDa on the RapifleX. This peak list was used to align the sample to a common m/z axis to allow direct comparison across different samples.

Although the peak fitting clearly does a good job of fitting the strongest features, there are some peaks (such as near 7.75 kDa or 7.85 kDa shown in Figure 5b) that are not fit, because they were not identified by the peak fitting algorithm with the specified parameters. Because each sample will have a different relative abundance of proteins, the intensity of individual peaks will vary across samples. This means that, if a peak is not detected by the peak finding algorithm for a given sample, either because the peak is low intensity, too close to a more prominent peak, or not present in the sample, it may be detected in a different sample. We assume that most proteins are present across different samples albeit at very different concentrations. To determine the set of potential peaks, in blood-based samples, we could detect the lists of peaks from the qualification set of 40 different samples and from the reference sample (measured with 220 unique preparations and acquisitions as described in Section 4.2) which were merged into a master list of unique peaks, resulting in a total of 1657 peaks for the RapifleX and 1256 peaks for the SimulTOF100 instruments.

Accurate peak intensities can be calculated by fitting the pre-defined peak shape function, described in Section 2.1, to each peak in the master peak list, yielding a semi-quantitative feature value for each peak (“Standard” feature value). Here, we utilize the fitted peak amplitude, $A_{0}$, as the standard feature value, but we note that other choices of the feature value, such as the area under the fitted peak, could also be used. The result of the fit of all peaks is shown in Figure 5c for the same acquisition and m/z range, as was shown in Figure 5b.

### 2.3. Reproducibility

**Figure 6:**
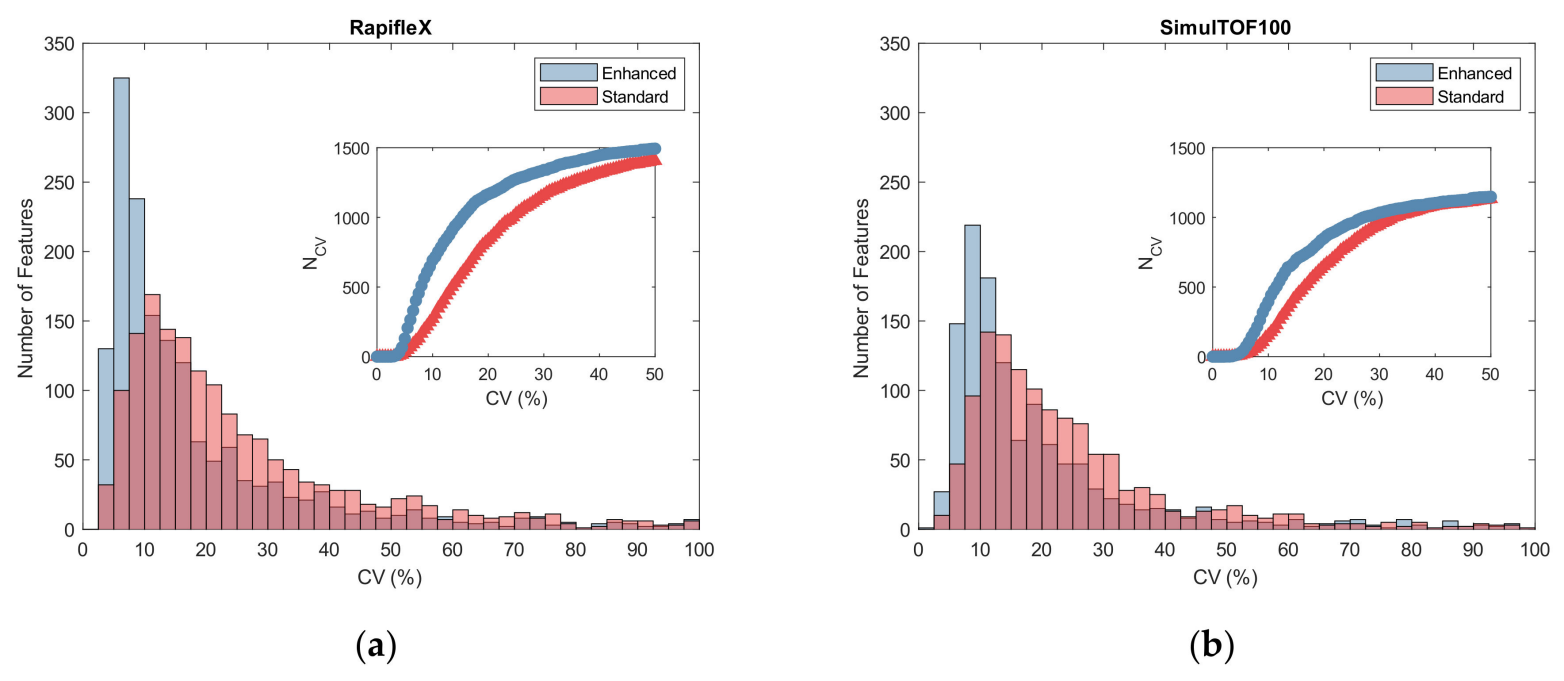
Reproducibility of feature values for a single sample over 20 preparations and acquisitions collected on the (**a**) RapifleX and (**b**) SimulTOF100. Histograms of CVs for standard (red) and enhanced (blue) feature values are shown in the main plot. The inset shows the cumulative CV distribution, $N_{CV}$, for the standard (red, triangles) and enhanced feature values (blue, circles) (only CVs up to 50% are shown for clarity).

To determine the reproducibility of the spectral processing and the resulting feature values, Deep MALDI spectra obtained from 20 replicate preparations of the reference sample were processed and the variations in the calculated feature values were compared. The coefficient of variation (CV) was measured for each feature using the standard feature values of the fitted peaks, as shown in red in Figure 6. We found that the reproducibility could be further improved by including the information in the bumps. For each peak, the “Enhanced” feature value was calculated as the sum of the fitted fine structure peak amplitude and the intensity of the bumps spectrum at the same m/z location. The CV distribution for the enhanced feature values for all peaks in the master peak list is shown in blue in Figure 6.

The improvement in reproducibility is further shown by the traces in the insets of Figure 6 which show the cumulative CV distribution, $N_{CV}$, for the two methods of analysis, where

(5) $$N_{CV}\left(x\right)=P\left(CV\leq x\right),$$

and $P\left(CV\leq x\right)$ is the probability that the CV is less than or equal to x. The enhanced feature value trace shows substantially more features with CVs that were lower than the standard features for the entire range. For example, for the RapifleX spectra, using enhanced feature values, there are 1000 feature with CV < 15.26%. However, while using standard feature values, there are only 594 features. In the following analysis, we restrict ourselves to the feature values calculated using the enhanced approach.

**Figure 7:**
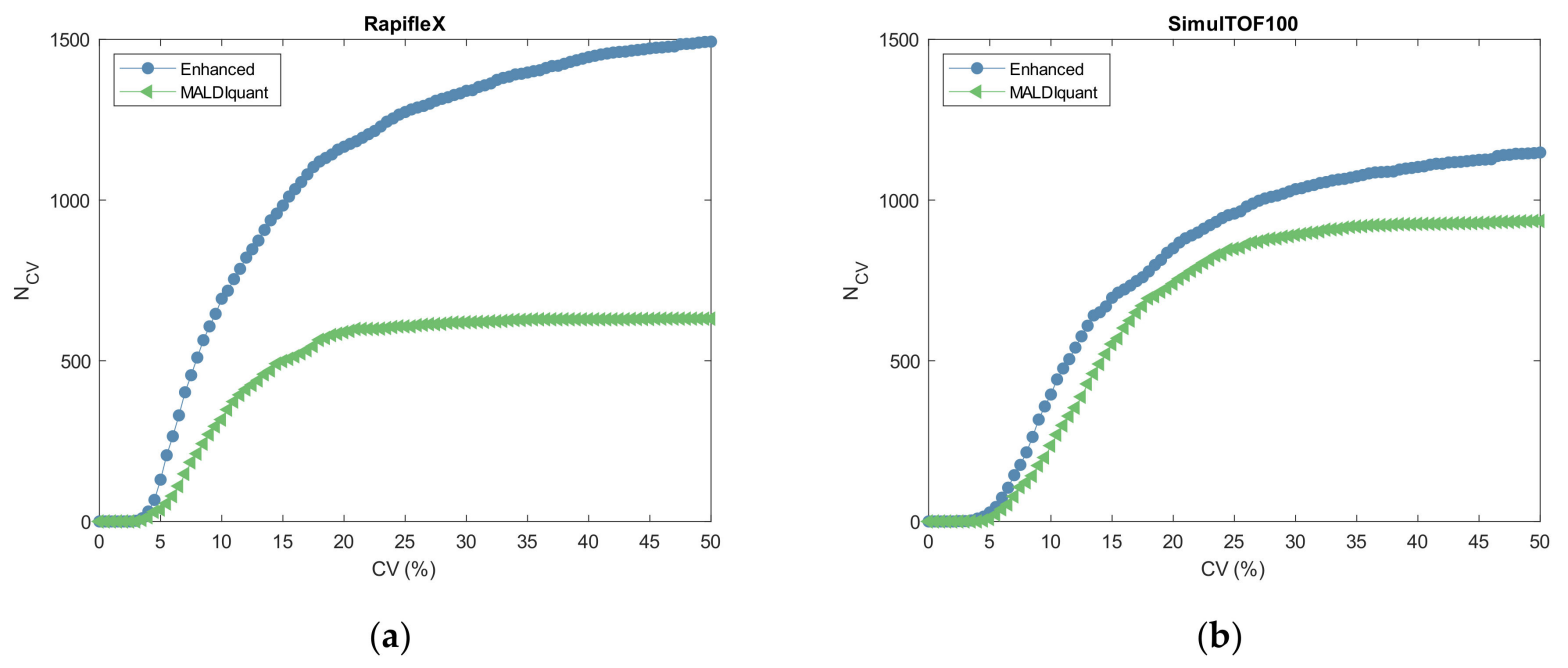
Comparison of reproducibility of our approach to a published method, MALDIquant. Cumulative CV distribution, $N_{CV}$, for the same Deep MALDI spectra analyzed with the presented methods using enhanced feature values (blue, circles) and with MALDIquant processing (green, triangles) for: (**a**) RapifleX and (**b**) SimulTOF100 acquisitions. Only CVs up to 50% are shown for clarity.

To compare the reproducibility of our processing to that of a commonly used software package, we analyzed our Deep MALDI spectra using the MALDIquant software package [24]. To maintain consistency with our method of creating a master peak list, we utilized the built-in functionality of MALDIquant to bin peaks over the identical 220 spectrum set to generate its own master peak list. The total number of peaks to fit was determined using the median absolute deviation (MAD) method with a SNR cutoff of 2 [22]. We intentionally chose a low SNR to select as many features as possible. Due to slight variations in peak positions not being numerically identical, we binned the peaks with a 0.002 Da tolerance, which resulted in 635 features for the RapifleX acquisition and 947 features for the SimulTOF100. CVs were calculated for the 20 replicate measurements of the reference sample and the comparison with the results from our processing methods, as shown in Figure 7.

### 2.4. Association with Biological Processes

It is important that the features represented here not only present reproducible data, but also are also biologically relevant. We calculated the association of each feature with 23 biological processes using protein set enrichment analysis (PSEA) [6,29]. This commonly used bioinformatics tool determines the association between a measured quantity, and, in our case, a mass spectral feature and a biological process by assessing the correlation between the measured quantity and the abundances of a set of known proteins related to the biological process. Table 2 shows the total number of features that were determined to be associated with each biological process with *p*-value of association < 0.01 and a false discovery rate (FDR) of < 5%.

## 3. Discussion

The goal of our processing methods is to better characterize complex MALDI-TOF spectra by improving peak detection and quantification. Because common peak detection approaches often perform poorly for clustered peaks [27,28], we utilized the method of spectral convolution to select peaks. Quantitation of peak intensity is also difficult to accurately determine when the peak of interest is part of a clusters of peaks. One cannot simply take the maximum intensity at the peak location because the tails of adjacent and overlapping peaks will add to the overall intensity at that location. Due to this complication, previous work would define the entire cluster as a single feature that spanned a spectral range instead of decomposing the cluster into individual peaks [6,12]. By accurately fitting the clusters, each individual peak intensity can be accurately determined without the influence of adjacent peaks. By implementing these ideas and utilizing the often-neglected information in the component of the spectra that varies more slowly with m/z (the bumps), we can detect more peaks with improved reproducibility than the traditional processing method tested [24]. The improved characterization can also lead to a better understanding of the direct biological implications of different features in our spectra.

### 3.1. MALDI Peak Shape Analysis

The m/z dependence on the peak shape is due to inherent protein properties (isotopic distribution) and the instrument response function (IRF). For any individual protein, the peak shape that we observe in a spectrum is a convolution of the isotopic distribution of the protein with the instrument response function. As proteins get larger, we expect to see a wider isotopic distribution [33]. An estimation of the peak width change with mass is shown in the Supplementary Materials, Figure S5, based on proteins composed of the fictional amino acid known as averagine [33]. Similarly, the mass spectrometer is known to have a variable IRF over wide mass ranges that results in wider features further from the optimal (tuned) mass range [28,34,35]. The change in trend from linear to quadratic shown in Figure 3b is likely due to a change in the IRF.

The IRF is a difficult parameter to determine [28,34,35,36]; thus, for this work, we opted to use an empirical fit. We note that, if the IRF could be carefully measured, it would be possible to get higher-resolution spectra by deconvoluting the observed spectra with the IRF and the isotope distribution [28]. Such information could be useful in better determining component parts of the bumps or perhaps eliminate the bumps altogether.

### 3.2. Peak Detection and Feature Value Determination

Traditional fitting methods often rely on simply removing the broad structures (bumps) during background subtraction, resulting in only sharp features (fine structure) [37,38]. These broad features originate from real biological content that is unresolved (see Figure 2) and, thus, potentially valuable information is lost when the bumps are overlooked during background estimation and subtraction. During the spectral analysis presented here, an individual spectrum was decomposed into three separate components: the background, the fine structure, and the bumps. By maintaining a slowly varying background, we are able to extract and analyze the bumps spectrum which was shown to improve quantitative reproducibility.

Although our methods show improved quantification of highly reproducible features, we do note that our method of processing is computationally slower (~3 min/spectrum) than the MALDIquant software (several seconds/spectrum) [24]. This is, in part, due to the high level MATLAB language, which could be sped up with a faster language, but it is also due to the differences in our peak detection algorithms. In the present work, we enhance our peak detection by convoluting the spectrum with the asymmetric peak shape that we defined for each instrument. Although this is a computationally intensive task, the convolution sharpens features and effectively filters the noise, which allows for more accurate detection of low intensity peaks. The MAD peak detection algorithm that we utilized in the MALDIquant analysis is one of the most commonly used peak-finding algorithms [22,23,24]. It simply finds local maxima and only selects those that are above the SNR cutoff. Because of the convolution we used, for the RapifleX data, we were able to find a total of 1657 peaks while using a SNR cutoff of 10, while the traditional MAD method used in the MALDIquant processing only found 635 features with a SNR of 2.

To further evaluate our algorithms, we processed an additional 220 sample preparations on another mass spectrometer (SimulTOF100). The spectra acquired on the SimulTOF100 were processed using the presented methods, and we found 1256 unique features (see Supplementary Materials Figure S3 and Table S1 for peak shape analysis on the SimulTOF100). The Deep MALDI spectra collected on the SimulTOF100 were also analyzed using MALDIquant, which found 947 features. Although the MALDIquant processing appeared to do better with this instrument, we still find that our processing methods produce a greater number of highly reproducible features.

### 3.3. Reproducibility

Our methods show an improvement over current traditional processing techniques, as tested by the MALDIquant software package [24]. For both sample sets run on the RapifleX and SimulTOF100 instruments, we defined and characterized a greater number of features (with enhanced feature values) with smaller CVs with the presented processing than with the MALDIquant software package. Because the spectral processing methods presented here show improvements across multiple instruments, we expect a similar performance using alternative sample preparation methods, such as utilizing depletion methods to improve the detection of low-abundance proteins [39,40].

Due to the large number of highly reproducible features, diagnostic tests could be created to stratify and classify patients into different groups to predict patient outcome based on this processing method. Multiple tests are currently being developed using the methods presented here which we believe will provide useful and actionable results for physicians and patients.

### 3.4. PSEA

Gene set enrichment analysis (GSEA) is a commonly used tool in bioinformatics that associates a measured quantity (for example, gene expression) with a biological process by finding patterns of association across a set of genes known to be related to that process [41]. Using a similar approach, we can associate our Deep MALDI features with biological processes in a protein set enrichment analysis [29,42].

In our previous work on the development of Deep MALDI [6], we demonstrated an improvement in the number of features associated with biological processes with ab increasing number of laser shots. The direct comparison (using the same number of laser shots generated by the Deep MALDI spectra, as well as the same *p*-value cutoff and FDR) to the previous work is shown in Table 2. The work by Tsypin et al. [6] was run on a SimulTOF100, so we can see a clear improvement in the number of associated features with our processing. The methods and procedures presented here show a substantial increase in the number of associated features in nearly all the biological processes investigated.

## 4. Materials and Methods

### 4.1. Serum Samples

A total of 40 serum samples (“qualification set”), derived from the blood of lung cancer and colorectal cancer patients, were purchased from Discovery Life Sciences, Inc. (Huntsville, AL, USA). A reference sample was created by pooling equal volumes of serum obtained from ten healthy individuals, also purchased from Discovery Life Sciences, Inc. The 100 serum samples collected from patients with non-small cell lung cancer used for association with biological processes via protein set enrichment analysis (“PSEA set”) were purchased from Oncology Metrics, LLC (Fort Worth, TX, USA) and Discovery Life Sciences Inc. (Huntsville, AL, USA). All samples were collected under ethics-approved protocols, according to the requirements of Discovery Life Sciences Inc. and Oncology Metrics LLC, and were stored at −80 °C.

### 4.2. Sample Preparation

Serum samples were thawed and 3-μL aliquots of each sample were spotted onto a serum card (GE HealthCare, Chicago, IL, USA). The spots were allowed to dry for 1 h at ambient temperature after which the whole serum spot was punched out from the underside with a 6-mm skin biopsy punch (Acuderm, Fort Lauderdale, FL, USA). Each punch was placed in a centrifugal filter with a 0.45-µm nylon membrane (VWR, Randor, PA, USA). In cases where the serum spots had spread outside the 6-mm diameter, the section where serum was visible was excised and added to the tube containing the 6-mm punch. To the centrifugal filter containing the punch, 100 μL of HPLC grade water (VWR, Randor, PA, USA was added. The punches were vortexed gently for 10 min and then spun down at 14,000 rcf for two minutes. The flow-through was removed and transferred back on to the punch for a second round of extraction consisting of vortexing gently for three minutes and spinning down at 14,000 rcf for two minutes. Finally, 20 μL of the filtrate from each sample was then transferred to a 0.5-mL Eppendorf tube. All subsequent sample preparation steps were carried out in a custom designed humidity and temperature control chamber (Coy Laboratory). The temperature was set to 30 °C and the relative humidity at 10%.

An equal volume of freshly prepared matrix (25 mg of sinapinic acid per 1 mL of 50% acetonitrile mixed with 50% water plus 0.1% TFA) was added to each 20-μL serum extract and the mix was vortexed for 30 s. The first three aliquots (3 × 2 μL, for SimulTOF100) or five aliquots (5 × 2 μL, for Rapiflex) of sample–matrix mix were discarded into the tube cap. Eight aliquots of 2-μL sample–matrix mix were then spotted onto a stainless steel MALDI target plate (Bruker, Billerica, MA, USA and SimulTOF Systems, Marlborough, MA, USA for spectra acquisition on the RapifleX and SimulTOF 100, respectively). The MALDI plate was allowed to dry in the chamber before placement in the MALDI mass spectrometer.

For the work on generating a master peak list and reproducibility a total of five replicate batches of the qualification set (40 serum samples) were analyzed for each mass spectrometer. Each batch consisted of the 40 serum samples with an additional four preparations of reference sample used as controls, with two preparations spotted at the start and two at the end of the batch. This resulted in a total of 220 spectra per mass spectrometer (5 × 40 samples in the qualification set and 20× of the reference sample).

Samples for the PSEA set were run in batches of up to 44 samples with an additional four preparations of the reference sample used as controls, with two preparations spotted at the start and two at the end of the batch for each mass spectrometer.

### 4.3. Mass Spectra Acquisition

#### 4.3.1. RapifleX

MALDI spectra were obtained using a RapifleX MALDI-TOF mass spectrometer (Bruker, Billerica, MA, USA). The instrument was operated in a positive ion mode, with ions generated using a frequency-tripled Nd:YAG laser emitting at 355 nm with a laser repetition rate of 5 kHz. Spectra were acquired in the 3 kDa to 30 kDa m/z range with a sampling rate of 0.63 Gs/s. External calibration was performed using the following peaks in the spectra generated from the reference samples included on every target plate (or batch): m/z = 3320, 4158.7338, 6636.7971, 9429.302, 13,890.4398, 15,877.5801, and 28,093.951. From each spot, 100 raster spectra were collected, totaling 800 raster spectra per sample. A raster spectrum is an average over 800 laser shots measured across a single spot.

#### 4.3.2. SimulTOF100

MALDI spectra obtained using a SimulTOF100 MALDI-TOF mass spectrometer (SimulTOF Systems, Marlborough, MA, USA). The instrument operated in the positive ion mode with ions generated using a 349-nm, diode-pumped, frequency-tripled Nd:YLF laser operated at a laser repetition rate of 0.5 kHz. Raster spectra were acquired in the 3 to 75 kDa m/z range (only the range from 3 to 30 kDa was used in this analysis) and were ‘hardware averaged’ to contain 800 laser shots as the laser fires continuously across the spot while the stage is moving at a speed of 0.25 mm/s. External calibration was performed using the following peaks generated in the reference sample included on every target plate: m/z =3320, 4158.7338, 6636.7971, 9429.302, 13,890.4398, 15,877.5801, and 28,093.951.

### 4.4. Spectral Analysis

**Scheme 1:**
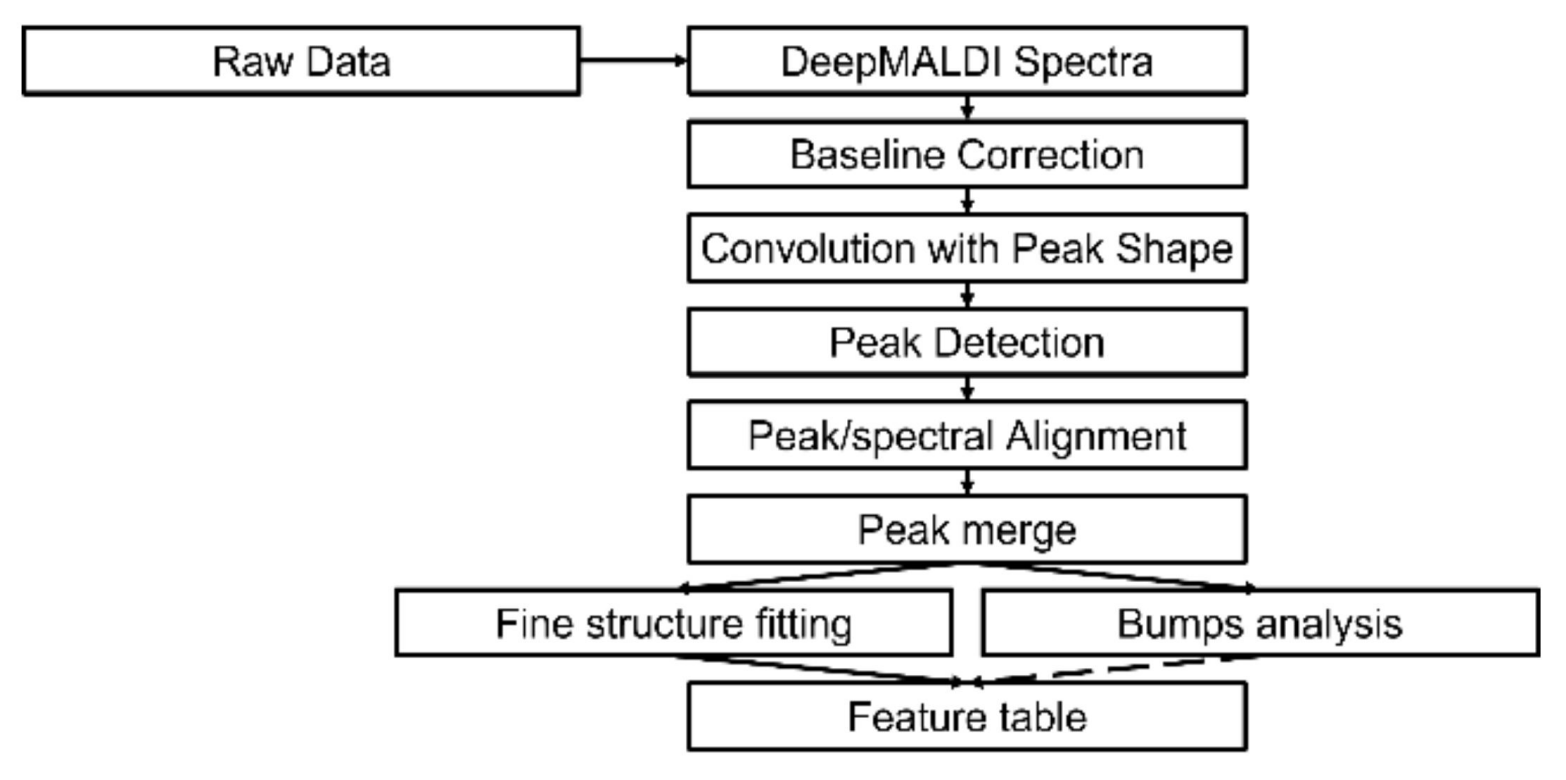
Spectral analysis workflow for mass spectrometer data using presented preprocessing methods.

The spectral analysis workflow is shown in Scheme 1 for processing of raw data through generation of a feature table or matrix (a list of feature values for each feature for each sample). Post-processing, such as normalization or corrections, can be performed on the table of feature values.

#### 4.4.1. Raster Averaging for Deep MALDI Spectra

To increase the number of observable peaks and to improve the SNR in the MALDI-TOF spectra, we employed the Deep MALDI raster averaging technique. For a complete description of this technique, we refer the reader to Tsypin et al. [6]. Briefly, each raster spectrum of 800 shots was processed through an alignment workflow to align peaks to a set of internal alignment points (Tables S2 and S3). Peaks were detected in each raster spectrum with a SNR cut-off > 3.0. The identified peaks for a raster spectrum were then used together with the set of predefined alignment peak positions to establish the coefficients in a second-order polynomial (in m/z) that was used to transform the m/z values of this raster spectrum. For successful alignment, we required a minimum of 20 detected peaks, with at least 13 peaks useable for alignment, i.e., an un-aligned peak position within a fixed alignment tolerance (1500 ppm) of the alignment peak. A maximum shift of 15 Da was allowed at the lowest m/z alignment point.

Averages were created from the pool of aligned raster spectra that satisfied the alignment quality criteria. A random selection of 500 raster spectra, without replacement, were averaged to create a final analysis spectrum of 400,000 shots for each sample.

#### 4.4.2. Background Estimation

The spectrum background was calculated using a stiff Eilers’ estimation ($\lambda_{1}=10^{11}$, $\lambda_{2}=10^{4}$, p=0.001) [31,32]. Briefly, the Eilers’ estimation is an asymmetric least squares fitting that penalizes positive and negative deviations separately, allowing for accurate estimation of the background.

#### 4.4.3. Fine Structure and Bumps Determination

The stiff background does not overfit the spectra and subtraction from the spectra results in sharp features that still are sitting on top of broad features, as seen in Figure 2 and Figure 4. Both the bumps and the sharp peaks may contain useful information as to total protein content in the sample but need to be treated separately to ensure accurate estimation of feature values.

A second, aggressive background was calculated to fit both the background and bumps with Eilers’ parameters $\lambda_{1}=10^{6}$, $\lambda_{2}=10^{2}$, and p=0.001. The difference between the spectrum and the aggressive background results in the fine structure, which contains the information of the sharp peaks on a flat background. The bumps were defined as the difference between the aggressive background and the stiff background.

#### 4.4.4. Peak Detection

Peak candidates to be fit were estimated using a peak finding algorithm based on the convolution of the fine structure with the peak-shape defined in Section 2.1. Peak candidate locations were estimated using the MATLAB function *islocalmin* on the second derivative of the fine structure, with a prominence window equal to the width of the FWHM of a peak and a minimum separation of peaks equal to 1/4 of the peak FWHM at the m/z location. These candidates were only fit as peaks if the SNR was greater than 10 and if the candidate was not being influenced by adjacent peak candidates. The signal was simply the intensity of the signal at the m/z point, while the noise was measured as the deviation in the signal from the average, as estimated by a Gaussian-smoothing window the size of the peak width.

A given peak was determined to be influenced by an adjacent peak if the peak centers were within half a peak width of each other or if either peak intersected the other at more than 10% of the maximum amplitude. Peak candidates with SNR > 10, which were not found to be influenced by an adjacent peak, were fitted to a single asymmetric Gaussian to get precise peak position and amplitude. Peak candidates with SNR > 10, but which were determined to be influenced by adjacent peak candidates, were assigned to be part of a cluster. The multiplicity of a cluster, *N*, is defined as the number of peak candidates that are influenced by at least one other member of the cluster. Clusters were fitted simultaneously by *N* asymmetric Gaussians (i.e., a doublet would be fit to *N* = 2 asymmetric Gaussians and a triplet would be fit to *N* = 3 asymmetric Gaussians). This method of fitting allows for accurate determination of the m/z position as well as peak intensity of all *N* peaks in the cluster. Peaks with a SNR < 10 were not considered for alignment purposes or merging into the master list.

A list of all peak locations for each sample was later merged into a master list of all measurable peaks from a wide range of multiple samples, as described in Section 4.6 below.

#### 4.4.5. Spectral Alignment

Spectra were aligned to a common m/z axis to ensure accurate feature (peak) intensities across samples. Alignment was performed by minimizing the variation in peak positions (m/z value from the peak fitting) for the sample with respect to the pre-specified alignment points (Tables S4 and S5). The m/z axis was rescaled using a second-order polynomial in m/z. Only peaks in the 80th percentile of SNR were used for alignment and were weighted inversely to their location in m/z, to account for the greater weights a simple linear regression gives to instances at higher m/z. A peak was determined to be alignable if its spectral position was within half of a peak width (as defined at the m/z position) of the nearest alignment point. To account for variations over the large range in m/z, we split the alignment range into four sub-ranges, as shown in Table S6.

To ensure the high-quality data we require, only fits with at least five alignable features in each region were used. Spectra that failed to align were not used in further determination.

#### 4.4.6. Feature Value Determination

The fine structure was fitted to 1657 (1256) asymmetric Gaussians at the specified m/z positions (see Section 4.6) to extract the peak intensity for the RapifleX (SimulTOF100). Isolated peaks were simply fitted to a single asymmetric Gaussian, while peaks that were part of a cluster were simultaneously fitted to *N* asymmetric Gaussians, where *N* is the multiplicity of the cluster. By fitting the entire cluster simultaneously, we ensured accurate peak amplitude measurements for peaks with significant overlap. A subtle, yet important, point to note is that we only fitted the intensity of each peak while keeping the m/z position and width parameters fixed (unlike in Section 4.4.5 above, where the m/z position was also fitted). Because the spectra were aligned in the previous step, we did not need to fit the m/z position and we could fit all features, even including those whose SNR was under 10. This results in our ability to accurately fit peaks whose intensity ranges over 3.8 orders of magnitude. A preliminary “Standard” feature value, characterizing the magnitude of a peak, was defined as the fitted peak amplitude. The preliminary feature value was further modified by adding the bump intensity at the m/z location to determine the “Enhanced” feature value. Mathematically,

(6) $$FV_{E}\left(m\right)=FV_{S}\left(m\right)+Bumps\left(m\right),$$

where m is the m/z location, $FV_{i}\left(m\right)$ for i=S,E is the feature value for “Standard” or “Enhanced”, respectively, and $Bumps\left(m\right)$ is defined above in Equation (3).

#### 4.4.7. MALDIquant Analysis

The same set of 220 Deep MALDI spectra (as described for the qualification set in Section 4.2 above), used to determine our master peak list (see Section 4.6), were analyzed using the MALDIquant mass spectra analysis software as a methods comparison [24]. Deep MALDI spectra were transformed using the square root transformation method to minimize any variance from the mean. Spectra were then smoothed with the Savitzky-Golay-Filter (halfWindowSize = 10) [43] and the baseline was corrected with the Statistics-sensitive Non-linear Iterative Peak-clipping (SNIP) algorithm (iterations = 100) [44]. Spectra were total ion current (TIC) normalized and spectra were aligned using the “lowess” warping method (halfWindowSize = 20, SNR = 2, and tolerance = 0.002) [45]. Peaks were determined using the MAD method (halfWindowSize = 20, SNR = 20) [22] and similar peaks were binned with a tolerance of 0.002. Feature values and CVs were calculated using the 635 unique peaks determined by this method for RapifleX Deep MALDI spectra and 947 unique peaks for the SimulTOF100 Deep MALDI spectra.

### 4.5. Peak Shape Fitting

A total of 19 isolated peaks were fitted to asymmetric gaussians (Equation (1)). The peaks were selected as isolated peaks that spanned the m/z range (3 to 30 kDa). Only the top 75% intensity was fitted for peak width determination. The trends of the left- and right-HWHM were then fit to a linear trend in the low mass region (3 to 17 kDa) and a quadratic fit for the high-mass region (13 to 30 kDa), as described by Equation (2). The linear and quadratic fits were intentionally made to overlap to accurately determine the intersection of the two curves ($m_{int}$). The average peak shape trend parameters came from the average of 12 different preparations of the same reference serum sample measured over three batches.

To ensure there were no discontinuities while fitting the entire spectra the final $m_{int}$ for FWHM, $\sigma_{L}$ and $\sigma_{R}$ were calculated solely from the FWHM trend.

### 4.6. Merge Peak Lists

Two-hundred and twenty peak lists, determined in Section 4.4.4, were generated for five batches of the qualification set as described in Section 4.2. All the peaks were merged into a single list resulting in 1657 unique peaks for the RapifleX and 1256 unique peaks for the SimulTOF100 (Supplementary Materials Tables S7 and S8). The merged peak list was created by iteratively comparing the merged peak list (initially empty) with an un-merged list. Peaks from the un-merged list that had a peak center greater than 0.5× peak width away from adjacent peak centers in the merge list were added. Peaks with centers less than 0.5× peak width away from adjacent peaks in the merge list had their location averaged with the existing merge peak.

### 4.7. Reproducibility Analysis

A total of 20 replicate measurements, including sample preparation and spectra acquisition, of the reference sample were collected over five batches. Spectra were processed as described above and standard and enhanced feature values were calculated for each replicate. Feature values were normalized to the total feature value intensity for the sample. For each feature, the average feature value ($\bar{x}$), standard deviation ($\sigma_{x}$), and coefficient of variation ($CV=\sigma_{x}/\bar{x}$) were calculated.

### 4.8. Association with Biological Processes

We followed the procedure outlined in Grigorieva et al. [29] to determine the association of the identified mass spectral features with 23 biological processes using protein set enrichment analysis. The biological processes investigated included both those expected to be assessable in circulation of patients with cancer (e.g., acute phase response, acute inflammatory response, wound healing) and some processes designed as controls (behavior, cellular components of morphogenesis). Briefly, protein abundance for 1305 known proteins was obtained for the PSEA set of 100 serum samples using the aptamer-based 1.3 k SOMAscan assay (SomaLogic, Boulder, CO, USA) [46,47]. The subsets of the 1305 proteins known to be associated with each of the 23 biological processes were identified using database searches, as has been previously described in detail [29]. Deep MALDI spectra were acquired from the PSEA set using both the RapifleX and the SimulTOF100 mass spectrometers using the methods of Section 4.2 and Section 4.3. The spectra were processed, and the feature values for each sample are described in Section 4.4, Section 4.5 and Section 4.6. The Spearman correlation was calculated between each feature and each of the 1305 proteins across the 100 different samples. An enrichment score was generated for each of the 23 biological processes for each mass spectral feature using the method of Roder et al. [42] with 25 splits of the sample set to provide increased power to detect association with biological processes compared with the standard GSEA enrichment score [41]. We calculated the *p*-values of association between each feature and the biological processes by comparing the enrichment score to a null distribution generated by a random permutation of feature values across the sample set. Features with a *p*-value of association < 0.01 and a false discovery rate of 5% or less, as estimated by the method of Benjamini-Hochberg [48] for multiple comparisons across the 23 biological processes, were determined to be associated with a given biological process. A subset of 1516 features were used from the Rapiflex processing and 1138 features for the SimulTOF100. For comparison, Ref [6] used 298 features. These reduced feature sets were determined by removing features that are known to depend strongly on sample collection and processing details, i.e., features that are related to hemoglobin (and its multiply charged analogs) or to fibrinogen (whose spectral intensity often vary).

## 5. Conclusions

Here, we developed a novel method for analyzing MALDI-TOF spectra over a wide spectral range. We used our method to analyze spectra from multiple samples to find 1657 unique peaks with over 3.5 orders of magnitude intensity, compared to only 635 for the traditional processing methods [24]. Our use of a well-defined peak shape function for our instrumentation allows us to accurately detect a greater number of peaks, particularly among overlapping peaks. The use of peak shape also allows for accurate fitting of overlapping peaks for accurate peak amplitude measurements. When compared to a traditional processing method, we found a substantial increase in the number of highly reproducible features with low CVs. We further validated our processing by performing the same analysis on spectra collected on a mass spectrometer from a different manufacturer and showing improved detection and reproducibility. Finally, we analyzed a set of 100 samples with known protein variation to determine the number of features associated with biological processes. We found an increase in the number of features associated with biological processes compared to analysis of the same sample set with a different spectral processing method [6].

## Figures and Tables

**Table 1:** The average peak width parameters for the FWHM, and the left and right HWHM for the RapifleX.

	$a_{0}$	$a_{1}$	$c_{0}$	$c_{1}$	$c_{2}$	$m_{int}$
FWHM	2.878	5.89 × 10^−4^	5.764	−1.13 × 10^−3^	1.05 × 10^−7^	14,471
$\sigma_{L}$	1.374	2.55 × 10^−4^	−3.143	−9.53 × 10^−6^	3.99 × 10^−8^	
$\sigma_{R}$	1.504	3.33 × 10^−4^	8.907	−1.12 × 10^−3^	6.54 × 10^−8^	

**Table 2:** Number of features associated with each biological process with FDR of < 5% and a *p*-value of association < 0.01 for 400,000 shot spectra collected on the RapifleX and SimulTOF100 mass spectrometers using the processing and feature definitions presented in this paper. A comparison is made with the number of associated features obtained with the same 400,000 shot spectra collected on the SimulTOF100 mass spectrometer using an alternative processing and feature definition method [6]. The percentages show the proportion of all analyzed features that show an association with the biological process. Note the substantial increase in the number of associated features identified when the new processing feature definition method is used.

Biological Process	Associated Features		
	RapifleX	SimulTOF	Ref. [6]
Acute phase response	655 (43.2%)	460 (40.6%)	122 (40.9%)
Complement activation	619 (40.8%)	383 (33.7%)	70 (23.5%)
Acute inflammatory response	434 (28.6%)	317 (27.9%)	109 (36.6%)
IFN γ signaling/response	266 (17.5%)	147 (12.9%)	25 (8.4%)
Immune tolerance and suppression	227 (15.0%)	189 (16.6%)	31 (10.4%)
Wound healing	202 (13.3%)	160 (14.1%)	100 (33.6%)
IFN type 1 signaling/response	195 (12.9%)	82 (7.2%)	33 (11.1%)
Type 17 immune response	73 (4.8%)	82 (7.2%)	2 (0.7%)
Angiogenesis	54 (3.6%)	28 (2.5%)	2 (0.7%)
Response to hypoxia	42 (2.8%)	54 (4.7%)	7 (2.3%)
Cellular component morphogenesis	27 (1.8%)	6 (0.5%)	4 (1.3%)
Cytokine production involved in immune response	21 (1.4%)	31 (2.7%)	3 (1.0%)
Glycolysis	21 (1.4%)	36 (3.2%)	2 (0.7%)
Chronic inflammatory response	19 (1.3%)	23 (2.0%)	2 (0.7%)
Innate immune response	18 (1.2%)	30 (2.6%)	8 (2.7%)
Extracellular matrix organization	16 (1.1%)	4 (0.4%)	0 (0.0%)
Behavior	7 (0.5%)	2 (0.2%)	8 (2.7%)
Epithelial-mesenchymal transition	6 (0.4%)	4 (0.4%)	0 (0.0%)
NK cell mediated immunity	4 (0.3%)	11 (1.0%)	N/A *
T-cell mediated immunity	4 (0.3%)	5 (0.4%)	N/A *
Type 2 immune response	2 (0.1%)	11 (1.0%)	N/A *
B-cell mediated immunity	2 (0.1%)	6 (0.5%)	N/A *
Type 1 immune response	1 (0.1%)	1 (0.1%)	N/A *

* N/A indicates the number of features associated with the biological process was not published in Ref. [6].

## Data Availability

The data presented in the study are available in the supplementary materials. MATLAB code used for analysis is available on request from the corresponding author.

## Supplementary Materials

The following are available online, Figure S1: Representative, high-resolution unprocessed and processed RapifleX spectrum, Figure S2: SimulTOF100 Deep MALDI comparison, Figure S3: SimulTOF100 peak shape fitting, Table S1: SimulTOF100 peak shape parameters, Figure S4: Peak shape parameter stability, Figure S5: Isotopic contribution to peak shape broadening, Table S2: Raster alignment peaks for RapifleX, Table S3: Raster alignment peaks for SimulTOF100, Table S4: Deep MALDI Alignment points for RapifleX, Table S5: Deep MALDI Alignment points for SimulTOF100, Table S6: Spectral alignment ranges, Table S7: Peak list for RapifleX, and Table S8: Peak list for SimulTOF100. A .csv file of an example spectrum measured on the RapifleX and the calculated background, fine structure, and bumps spectral components is also available.

## Sample Availability

Samples of the compounds are not available from the authors.
