# Supplementary material for: Semi-Quantitative MALDI Measurements of Blood-Based Samples for Molecular Diagnostics
Source: Molecules. 2022 Feb 1;27(3):997. doi: 10.3390/molecules27030997 (PMC8840133; doi:10.3390/molecules27030997)
Supplement: Supplementary file 1 [file molecules-27-00997-s001.zip › SupplementaryFiles/SI_MoleculesPaper_211216.pdf]

Supplementary information for semi-quantitative MALDI measurements of blood-based samples for molecular diagnostics

Matthew A. Koc [1], Senait Asmellash [1], Patrick Norman[1], Steven Rightmyer[1], Joanna Roder [1], Robert W. Georgantas III[1], and Heinrich Roder [1]

[1]Biodesix Inc., Boulder, Colorado, United States of America

Figure S1 – Representative, high-resolution unprocessed and processed RapifleX spectrum

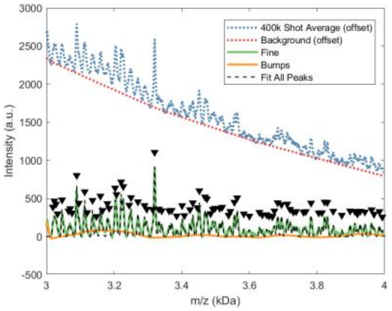

(a)

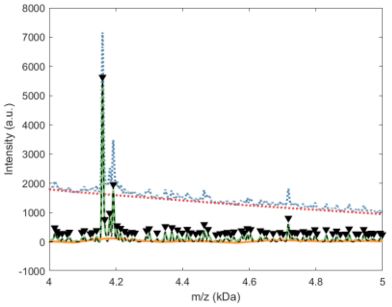

(b)

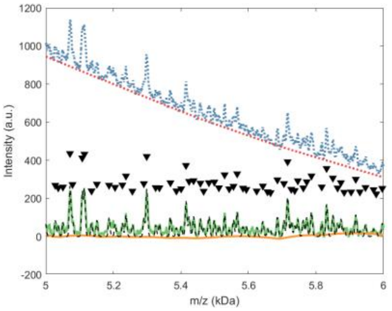

(c)

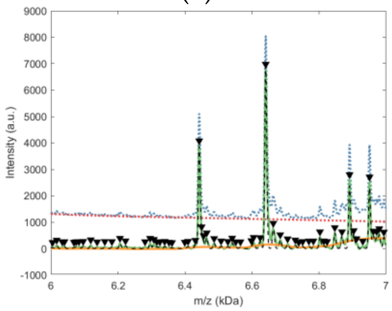

(d)

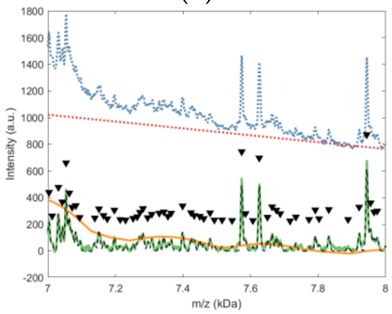

(e)

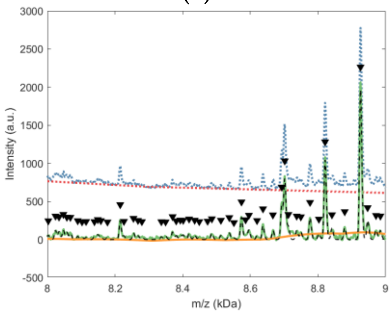

(f)

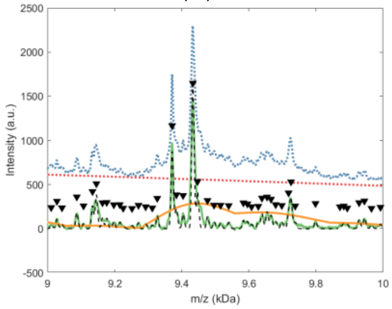

(g)

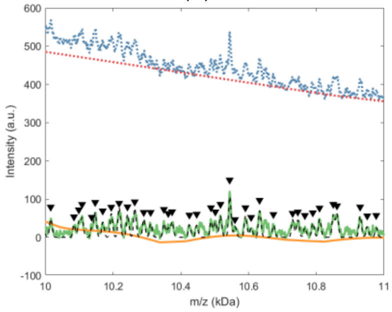

(h)

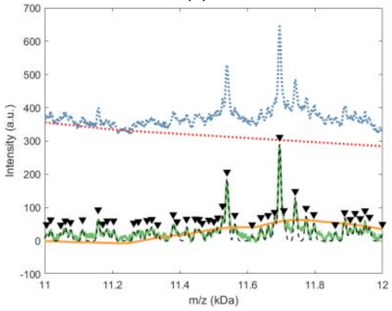

(i)

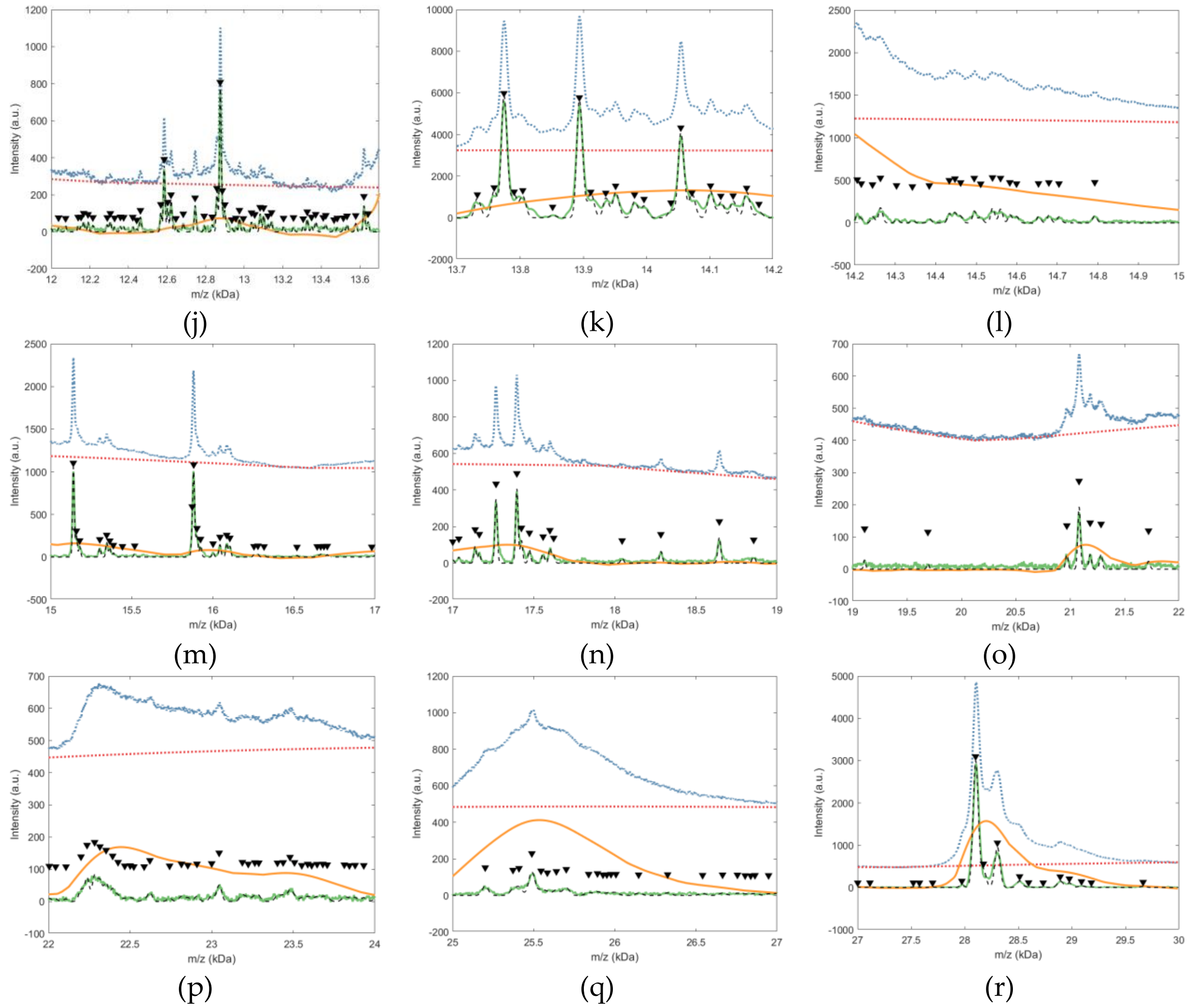

Figure S1. Example spectrum collected on the RapifleX. Each figure shows the unprocessed 400k Shot average Deep MALDI spectrum (blue, dotted), the background (red, dotted), the Fine structure (green, solid), the Bumps (yellow, solid), and the spectral fit (black, dashed) for all peaks (positions noted by the black triangles) for a given range. Panels a-r show the entire range from m/z = 3 to 30 kDa. For clarity the 400k Shot average and background were offset in intensity by a constant amount of: **a**) 6000 counts, **b-c**) 5000 counts, **d-j**) 4000 counts, **k**) 1000 counts, **l-m**) 3000 counts, **n-r**) 3500 counts. Figure 5a of the main text shows the full spectrum from 3-30 kDa without any offset for the 400k Shot average.

Figure S2 – SimulTOF100 Deep MALDI Comparison

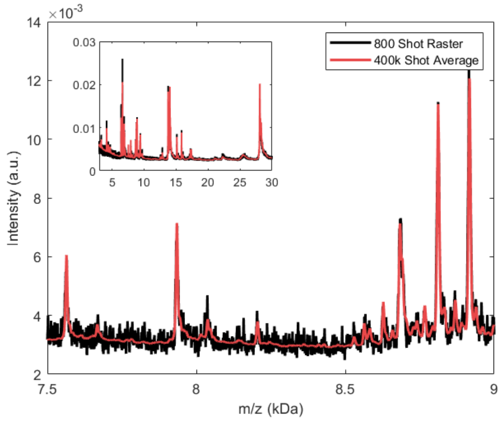

Figure S2. Example spectra collected on the SimulTOF100 of an individual raster spectrum (black) and a 400k shot Deep MALDI averaged spectrum (red) from 7.5 to 9 kDa m/z range. The inset shows the same spectra over the full 3 to 30 kDa range analyzed in this work.

Figure S3 – SimulTOF100 Peak shape fitting

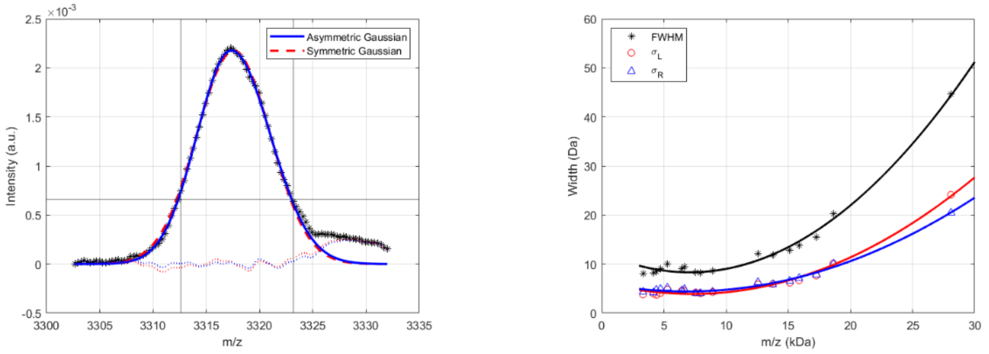

(**a**) (**b**)

Figure S3. Peak shape determination of SimulTOF100 MALDI-TOF spectral peaks. (**a**) Sample data (black stars) and peak fit to an asymmetric (blue-solid) and symmetric (red-dashed) Gaussian. Fit error is shown on the dotted lines. (**b**) Peak shape parameters as a function of m/z. Overall fitted trend are shown with solid lines and the linear (dashed) and quadratic (dotted) piecewise portions for σ_L and $\sigma_R$of the fits are extended past the trend range for reader visibility.

Table S1 – SimulTOF100 Peak shape parameters

Table S1. The average peak width parameters for the FWHM, left-, and right-HWHM for the ST100. Results were found to fit well to a single quadratic fit, so m_{int} was set to 0.

	a_0	a_1	c_0	c_1	c_2	m_{int}
FWHM	0	0	12.398	-1.15E-03	8.16E-08	0
σ_L	0	0	6.342	-6.69E-04	4.60E-08	0
σ_R	0	0	6.057	-4.85E-04	3.56E-08	0

Figure S4 – Peak shape parameters stability

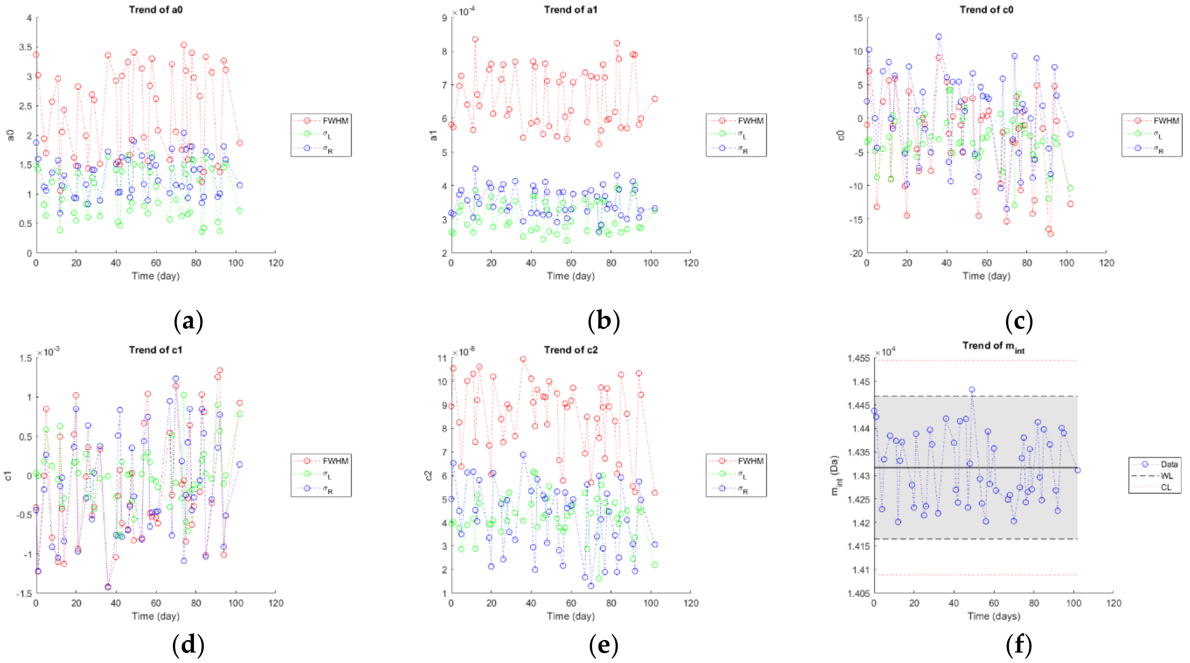

Figure S4. Trend charts for the RapifleX peakshape parameters over the course of >100 days of operation based on the same reference serum sample run on each batch. Trends are shown for (**a**) a_0, (**b**) a_1, (**c**) c_0, (**d**) c_1, (**e**) c_2, and (**f**) m_{int}. For a-e, the trends for the FWHM, σ_L, and σ_R are shown in red, green, and blue, respectively. The m_{int} trend in (f) shows the warning limit (WL, ± 2 standard deviations) and critical limit (CL, ± 3 standard deviations). All values show stable trends.

Figure S5 – Isotopic contribution to peak shape broadening

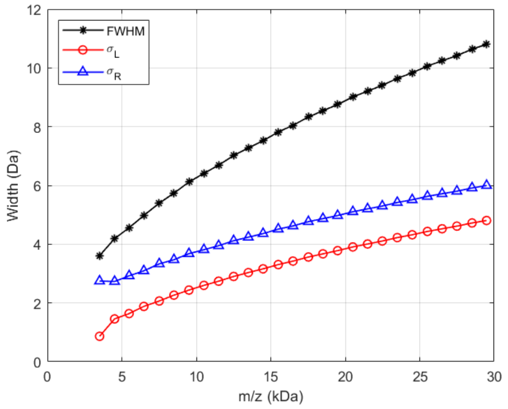

Figure S5. Calculated peak fitting trend due to isotope broadening. Isotopic distribution was calculated based off of the fictional isotope averagine described by Senko et al. [1] with an m/z spacing of 1 Da. Peaks were fit to an asymmetric Gaussian as described in the main text.

Table S2 – Raster alignment peaks for RapifleX

Table S2. Alignment points used for aligning individual rasters described in Section 4.4.1 on the Bruker RapifleX

m/z
3320.71
4160.27
4718.33
6442.52
6640.88
6891.39
7945.08
8692.87
8926.44
9432.89
11695.73
12876.73
13775.03
14054.90
15140.24
15882.10
17395.14
21084.41
22253.48
25493.56
28103.22
28305.38

Table S3 – Raster alignment peaks for SimulTOF100

Table S3. Alignment points used for aligning individual rasters described in Section 4.4.1 on the SimulTOF100.

m/z
3168.00
4153.48
4183.00
4792.00
5773.00
5802.00
6432.79
6631.06
7202.00
7563.00
7614.00
7934.00
8034.00
8206.35
8684.25

8812.00
8919.00
8994.00
9133.25
9310.00
9427.00
10739.00
10938.00
11527.06
12173.00
12572.38
12864.24
13555.00
13762.87
13881.55
14039.60
14405.00
15127.49
15263.00
15869.06
17253.06
18629.76
21065.65
23024.00
28090.00
28298.00

Table S4 – Deep MALDI Alignment points for RapifleX

Table S4. Alignment points for Deep MALDI spectra used in Section 4.4.5 of main text for the RapifleX.

3021.15	4201.71	5644.13	7281.70	9028.22	11274.86	13732.01	16996.95
3027.60	4213.91	5685.75	7296.51	9045.65	11289.37	13757.62	17038.22
3033.56	4224.39	5707.03	7308.22	9087.45	11305.17	13774.64	17086.11
3045.91	4247.45	5716.28	7329.84	9106.55	11317.76	13789.92	17138.33
3051.91	4258.68	5729.76	7343.32	9134.61	11349.10	13804.24	17165.77
3064.85	4271.49	5744.76	7355.51	9146.03	11383.25	13852.92	17267.10
3073.13	4291.38	5762.87	7365.61	9173.13	11395.64	13893.87	17289.33
3090.06	4299.95	5772.81	7385.10	9197.35	11419.74	13911.63	17395.29
3096.43	4323.09	5786.67	7399.43	9216.02	11451.58	13933.63	17422.56
3113.06	4348.38	5804.91	7423.53	9251.85	11463.34	13950.93	17472.12
3135.69	4367.94	5816.78	7433.81	9271.68	11487.50	13981.35	17492.10
3142.00	4386.39	5831.61	7451.07	9292.11	11504.47	13995.75	17557.54
3148.54	4399.47	5850.26	7462.35	9312.77	11525.68	14038.70	17600.64

3160.58	4412.97	5863.80	7480.65	9327.16	11539.71	14053.96	17622.29
3168.06	4419.60	5873.00	7492.67	9371.22	11553.68	14069.62	17648.88
3181.15	4426.38	5883.12	7515.24	9383.40	11565.03	14100.79	17814.32
3187.52	4441.39	5890.51	7547.29	9404.29	11578.78	14117.84	17881.35
3205.29	4451.54	5917.51	7574.16	9433.25	11617.09	14136.78	18025.83
3211.72	4465.03	5946.30	7584.66	9446.99	11638.27	14158.25	18049.81
3221.17	4474.13	5971.76	7594.47	9476.03	11660.49	14177.45	18283.40
3227.11	4480.40	5997.44	7607.30	9495.63	11681.35	14207.86	18588.29
3242.72	4498.52	6016.59	7626.50	9513.20	11695.14	14245.56	18644.85
3248.75	4514.34	6037.68	7650.95	9523.37	11709.15	14261.31	18806.46
3254.17	4531.93	6055.63	7664.30	9540.38	11741.86	14274.20	18857.24
3269.63	4544.77	6071.33	7677.59	9583.76	11773.43	14304.74	19044.18
3274.94	4555.04	6082.09	7687.85	9595.59	11796.63	14323.20	19110.35
3288.93	4577.30	6090.69	7720.16	9607.88	11840.68	14343.14	19576.12
3294.49	4584.58	6098.99	7735.25	9630.56	11887.94	14366.79	19694.60
3300.39	4594.38	6118.66	7748.32	9639.85	11903.76	14383.45	20336.22
3320.37	4604.61	6137.87	7774.24	9653.89	11918.21	14432.96	20546.91
3327.38	4633.96	6151.49	7792.95	9665.43	11932.48	14447.31	20970.56
3333.97	4650.29	6160.78	7804.62	9681.06	11948.23	14460.82	21083.65
3366.85	4672.15	6182.00	7825.42	9708.78	11962.76	14481.83	21182.29
3372.43	4685.61	6194.59	7833.29	9723.84	11976.16	14496.79	21294.96
3401.47	4693.19	6206.11	7844.80	9737.55	11995.65	14512.81	21479.45
3412.41	4704.52	6216.88	7877.31	9785.53	12009.79	14531.85	21723.22
3424.23	4718.12	6225.14	7889.84	9798.30	12036.68	14543.19	22204.76
3433.61	4728.42	6263.51	7922.31	9811.35	12062.47	14558.34	22241.26
3446.26	4737.64	6281.38	7944.81	9862.59	12075.62	14583.32	22278.76
3452.39	4745.54	6295.69	7955.60	9874.64	12124.02	14598.50	22315.64
3468.97	4762.90	6306.84	7965.98	9884.52	12138.17	14654.14	22355.21
3475.26	4783.40	6314.90	7976.16	9895.19	12152.00	14680.00	22397.00
3487.86	4797.93	6323.82	8003.86	9904.34	12166.94	14705.47	22618.74
3494.38	4821.18	6339.29	8024.35	9929.48	12191.20	14738.64	23047.67
3501.74	4831.30	6353.50	8036.02	9944.99	12293.03	14773.09	23187.44
3511.96	4852.28	6362.09	8047.67	9955.50	12306.91	14789.75	23476.56
3519.73	4863.40	6380.64	8057.95	9968.35	12347.24	14797.08	25202.40
3540.30	4874.62	6408.44	8068.79	9995.37	12360.25	14813.29	25405.93
3545.34	4891.53	6433.00	8088.79	10015.71	12394.94	14832.47	25488.17
3556.37	4899.87	6442.57	8129.39	10062.40	12423.77	14850.44	28104.40
3564.44	4918.87	6463.70	8139.33	10087.66	12462.49	14864.83	28303.83
3575.32	4929.41	6486.57	8149.57	10106.46	12516.12	14889.28	28511.15
3583.59	4944.46	6508.12	8158.47	10145.68	12568.45	14939.14	
3594.77	4952.17	6529.02	8171.73	10171.72	12585.08	14953.02	
3600.61	4967.16	6541.60	8180.84	10193.11	12602.91	14978.70	
3630.22	4974.07	6565.19	8215.45	10218.78	12620.67	15082.30	

3639.39	4993.87	6597.58	8235.04	10242.52	12643.56	15095.13	
3660.31	5009.51	6605.25	8245.76	10264.95	12667.24	15140.25	
3684.17	5029.13	6625.00	8255.63	10290.79	12683.97	15156.45	
3694.46	5040.16	6641.07	8267.75	10312.55	12700.88	15167.67	
3703.15	5052.66	6663.74	8279.35	10352.24	12716.54	15183.59	
3711.43	5066.93	6683.98	8288.67	10374.85	12733.47	15303.31	
3718.62	5073.69	6706.12	8323.88	10397.97	12746.00	15323.18	
3725.46	5080.86	6728.51	8337.72	10427.12	12792.09	15345.80	
3731.21	5108.76	6738.99	8351.76	10445.84	12809.35	15363.30	
3738.01	5114.95	6751.95	8371.43	10458.56	12833.26	15375.86	
3745.78	5121.39	6769.58	8396.79	10488.93	12858.60	15391.97	
3761.56	5137.96	6783.95	8419.57	10502.77	12876.01	15445.39	
3771.40	5152.73	6803.13	8437.67	10517.77	12888.05	15518.92	
3782.23	5187.40	6813.48	8458.49	10544.46	12898.42	15552.61	
3802.69	5196.03	6821.61	8473.84	10561.79	12911.05	15573.89	
3808.17	5202.96	6847.45	8485.69	10591.08	12957.51	15592.00	
3815.13	5227.45	6868.67	8497.53	10633.09	12974.62	15730.73	
3823.27	5237.61	6891.07	8514.39	10647.89	12997.00	15748.82	
3831.35	5256.37	6901.62	8539.15	10656.99	13039.27	15881.68	
3844.18	5283.23	6908.16	8574.33	10673.95	13057.19	15901.75	
3849.67	5291.20	6931.32	8588.73	10721.68	13081.90	15920.05	
3898.99	5299.06	6950.78	8596.95	10732.88	13100.33	15937.97	
3912.32	5309.84	6962.23	8620.23	10740.45	13123.68	16001.30	
3929.04	5327.16	6978.72	8637.79	10750.27	13141.37	16044.11	
3941.61	5336.56	6993.62	8667.44	10791.13	13164.64	16063.38	
3961.31	5356.17	7003.03	8693.53	10809.99	13181.68	16087.57	
3971.13	5370.79	7012.45	8702.54	10838.36	13208.15	16106.26	
3984.21	5381.42	7031.09	8713.60	10850.55	13255.63	16128.76	
3995.90	5400.98	7043.10	8736.60	10861.46	13279.00	16206.74	
4017.49	5414.80	7054.25	8750.37	10894.33	13306.56	16269.15	
4024.09	5423.49	7063.38	8776.97	10907.85	13327.51	16291.12	
4039.39	5437.53	7071.48	8803.21	10929.70	13348.60	16313.22	
4051.97	5458.17	7082.23	8821.79	10968.77	13369.26	16332.18	
4057.75	5482.65	7093.23	8831.07	10979.88	13383.25	16401.02	
4062.42	5500.96	7107.61	8841.66	11011.72	13403.06	16598.44	
4097.55	5511.57	7138.86	8853.12	11059.62	13421.34	16637.73	
4106.15	5519.60	7151.56	8879.19	11073.76	13439.43	16653.43	
4115.87	5529.56	7164.16	8899.71	11113.29	13521.62	16668.06	
4130.45	5555.50	7173.00	8909.16	11158.22	13534.90	16686.41	
4142.39	5566.59	7197.51	8926.58	11172.89	13551.37	16704.62	
4160.16	5581.93	7211.76	8946.08	11186.28	13578.46	16725.85	
4166.49	5592.75	7229.77	8970.72	11202.78	13620.93	16746.41	
4181.71	5599.42	7254.20	8984.40	11239.44	13642.32	16936.90	

4192.24	5637.00	7271.26	9008.13	11261.67	13687.63	16956.86	

Table S5 – Deep MALDI Alignment points for SimulTOF100

Table S5. Alignment points for Deep MALDI spectra used in Section 4.4.5 of main text for the SimulTOF100.

3044.72	5935.56	7914.00	9744.40	11761.76	14293.68	19145.52
3088.88	5964.08	7934.52	9788.19	11782.63	14307.40	19377.18
3111.46	5989.53	7945.12	9799.92	11794.33	14353.24	19479.06
3139.93	6007.60	7960.01	9851.39	11830.51	14369.72	19561.12
3157.23	6028.37	7975.93	9866.49	11860.90	14421.97	20398.48
3201.94	6063.19	7982.92	9874.01	11877.53	14438.10	20521.72
3218.95	6074.44	8015.97	9887.50	11908.77	14471.25	20545.26
3241.99	6082.52	8037.76	9919.54	11938.74	14482.31	20575.05
3265.78	6088.71	8047.53	9935.13	11950.57	14495.81	20596.89
3317.93	6109.69	8059.06	9949.97	11955.87	14528.47	20793.11
3369.26	6146.04	8090.29	9991.21	11960.80	14546.52	20828.38
3398.46	6173.16	8120.15	10008.17	12023.50	14580.01	20929.41
3423.25	6196.36	8124.38	10054.37	12051.47	14642.16	20960.51
3446.38	6211.35	8128.93	10062.69	12064.90	14667.83	21067.14
3467.74	6224.89	8140.57	10070.55	12108.94	14690.25	21166.56
3487.16	6286.19	8149.69	10078.25	12141.83	14779.99	21266.43
3556.05	6300.09	8160.00	10085.95	12156.60	14819.68	21370.23
3682.06	6311.96	8205.16	10097.96	12180.76	14840.68	21477.24
3690.97	6332.13	8216.01	10133.74	12205.82	14862.46	21603.69
3708.08	6389.91	8244.92	10139.87	12275.10	14878.18	21707.67
3725.99	6398.24	8256.75	10156.29	12280.09	14970.79	21757.35
3777.62	6433.23	8270.28	10180.01	12285.17	15128.18	21805.52
3820.58	6444.22	8313.81	10187.08	12296.79	15148.25	21855.12
3842.97	6479.13	8327.23	10206.30	12331.48	15168.16	21976.17
3892.70	6497.59	8339.94	10231.80	12345.47	15185.61	22187.17
3908.75	6521.24	8360.58	10252.81	12354.08	15226.63	22219.66
3955.74	6529.48	8369.34	10283.57	12366.37	15291.20	22254.36
3977.29	6537.53	8382.39	10301.25	12385.08	15311.71	22287.95
4013.72	6588.41	8387.29	10341.95	12410.84	15334.12	22320.74
4052.54	6619.31	8392.21	10362.99	12446.43	15352.26	22352.71
4101.26	6632.17	8411.43	10394.75	12453.12	15372.89	22373.30
4154.53	6641.72	8428.15	10413.73	12504.66	15390.07	22395.94
4186.14	6665.38	8443.47	10446.44	12557.44	15437.61	22432.72
4208.81	6675.10	8475.24	10477.40	12573.97	15494.79	22605.96
4245.76	6696.17	8487.70	10492.39	12590.78	15509.96	22805.01
4266.11	6721.15	8504.09	10507.93	12609.18	15539.96	23033.41
4291.81	6729.45	8529.05	10532.50	12632.46	15558.22	23172.57

4342.38	6738.46	8563.57	10583.98	12656.08	15575.08	23376.98
4362.15	6761.21	8581.60	10623.38	12672.18	15638.54	23465.14
4382.77	6794.67	8592.71	10633.63	12690.63	15740.99	23562.86
4406.40	6805.98	8627.05	10646.67	12733.82	15763.42	25188.48
4434.78	6837.92	8637.81	10667.81	12780.76	15869.10	25402.48
4458.55	6860.30	8657.44	10676.31	12796.85	15890.56	25476.63
4472.15	6881.27	8682.98	10685.22	12847.46	15913.58	25567.35
4509.02	6893.45	8691.95	10713.94	12856.03	15951.37	25680.23
4567.75	6903.47	8702.41	10720.89	12864.98	15969.99	27964.53
4587.20	6930.11	8727.52	10729.18	12876.73	16032.53	28094.06
4598.76	6940.93	8740.04	10739.05	12889.06	16075.01	28190.12
4627.20	6952.58	8765.95	10748.30	12900.86	16093.66	28298.85
4645.45	6969.23	8792.92	10781.58	12923.97	16115.59	28403.23
4675.96	6985.38	8811.27	10796.90	12948.40	16237.76	28509.99
4697.22	6993.11	8824.21	10836.63	12963.51	16257.43	28613.52
4711.44	7020.27	8868.62	10851.01	12981.28	16279.92	28717.54
4723.31	7034.09	8891.07	10879.87	13029.92	16299.27	28882.58
4757.32	7044.72	8916.20	10918.92	13045.59	16317.64	28975.40
4790.94	7055.43	8926.11	10927.89	13068.96	16468.51	29079.76
4814.34	7072.33	8959.69	10940.66	13088.49	16484.28	29188.59
4822.59	7084.67	8973.39	10951.01	13112.73	16503.34	
4842.83	7097.00	8997.87	10959.47	13131.07	16521.09	
4856.22	7104.72	9017.97	10968.90	13154.13	16542.17	
4892.05	7129.41	9034.14	10982.32	13169.64	16631.93	
4923.24	7142.09	9062.62	10994.68	13188.58	16653.99	
4938.13	7154.27	9075.46	11000.10	13244.08	16668.87	
4962.73	7188.46	9094.08	11005.48	13272.96	16687.40	
4983.68	7198.75	9124.36	11045.01	13294.47	16706.59	
4998.93	7211.95	9135.02	11059.16	13316.07	16716.31	
5042.97	7243.48	9149.34	11068.89	13335.98	16733.17	
5067.09	7260.06	9159.45	11080.28	13359.32	16832.05	
5104.71	7271.89	9168.43	11102.09	13372.18	16853.97	
5142.67	7284.74	9173.16	11147.95	13391.06	16926.89	
5174.26	7296.40	9179.89	11190.33	13410.25	17023.67	
5186.12	7319.71	9203.62	11254.59	13507.96	17125.45	
5196.40	7332.72	9241.30	11266.55	13524.95	17151.24	
5222.80	7344.95	9260.61	11288.73	13566.66	17253.66	
5248.89	7357.12	9282.93	11302.10	13588.57	17381.91	
5289.54	7389.29	9288.00	11310.88	13608.90	17402.70	
5327.21	7414.58	9292.58	11340.60	13630.13	17460.49	
5360.81	7422.92	9316.22	11351.91	13661.65	17588.89	
5406.75	7437.37	9351.96	11370.65	13675.23	17608.47	
5417.52	7443.39	9360.47	11381.49	13723.24	17640.31	

5429.34	7470.13	9373.18	11389.72	13746.84	17667.12	
5451.66	7504.48	9393.47	11394.52	13763.08	17799.31	
5474.62	7564.13	9423.18	11401.71	13779.91	17825.51	
5491.76	7573.44	9436.62	11411.35	13792.41	17874.96	
5520.63	7586.08	9463.38	11441.87	13843.34	18015.95	
5557.89	7611.49	9482.49	11453.10	13866.55	18039.97	
5573.14	7645.29	9497.43	11480.33	13882.22	18168.31	
5587.43	7653.28	9506.22	11487.75	13898.85	18269.61	
5601.63	7667.46	9531.58	11495.22	13939.30	18443.37	
5633.13	7677.18	9573.57	11515.72	13974.77	18474.64	
5707.46	7723.68	9589.70	11528.89	14041.44	18498.85	
5731.39	7738.26	9595.56	11558.90	14062.78	18574.91	
5750.99	7768.22	9624.10	11584.71	14089.45	18629.87	
5762.75	7782.37	9631.79	11604.03	14105.58	18726.98	
5777.17	7811.88	9644.90	11627.03	14140.60	18757.68	
5793.26	7821.14	9668.66	11649.75	14151.56	18795.17	
5822.22	7833.40	9699.15	11683.77	14168.60	18831.87	
5840.19	7853.87	9714.05	11722.81	14194.32	18856.22	
5864.02	7867.06	9724.83	11731.57	14247.22	18894.93	
5908.07	7879.53	9734.39	11747.55	14258.17	19093.63	

Table S6 – Spectral alignment ranges for RapifleX and SimulTOF100

Table S6. Spectral alignment ranges. The different ranges were determined visually based on where there appeared to be a spacing in the detected peaks.

	RapifleX		SimulTOF100	
	Begin m/z	End m/z	Begin m/z	End m/z
Range 1	3000.0	5167.9	3000.0	4998.9
Range 2	5167.9	9839.4	4998.9	9799.9
Range 3	9839.4	16173	9799.9	16116
Range 4	16173	30000	16116	30000

Table S7 – Peak list for RapifleX

Table S7. Peak list for the RapifleX consisting of 1657 peaks.

3015.81	4130.20	5482.45	7013.44	8745.53	10679.34	12776.21	15400.33	18932.00
3021.69	4133.82	5500.27	7018.84	8749.87	10683.92	12792.48	15413.60	18983.91
3027.40	4142.23	5504.96	7025.13	8759.95	10690.13	12809.35	15423.84	19013.54
3033.70	4146.87	5512.62	7030.77	8765.56	10703.41	12833.46	15441.70	19049.46
3046.40	4153.56	5521.33	7043.02	8772.36	10711.11	12846.30	15451.03	19068.76
3051.06	4160.02	5529.40	7054.20	8777.09	10721.46	12858.19	15465.03	19090.87
3054.47	4166.29	5535.75	7063.93	8791.56	10730.34	12866.18	15477.94	19112.66

3059.82	4170.81	5542.99	7071.75	8803.38	10739.81	12876.15	15491.90	19132.40
3069.11	4176.20	5550.14	7082.33	8821.59	10745.93	12888.09	15506.40	19156.89
3072.88	4181.73	5555.70	7093.66	8832.20	10767.90	12898.08	15520.50	19169.91
3076.49	4188.76	5559.45	7098.79	8841.99	10774.53	12912.28	15534.83	19196.85
3089.80	4192.15	5566.71	7106.36	8847.42	10782.35	12926.02	15551.18	19247.91
3096.06	4198.22	5582.39	7118.26	8853.36	10790.86	12937.82	15571.97	19283.01
3100.22	4202.89	5593.56	7124.66	8864.65	10810.11	12957.16	15591.70	19309.63
3112.88	4213.92	5599.78	7139.14	8879.24	10822.66	12974.87	15609.08	19324.10
3117.13	4218.08	5606.53	7151.57	8899.15	10836.94	12995.89	15618.07	19337.12
3120.77	4225.80	5612.08	7159.42	8908.52	10849.74	13007.57	15634.43	19393.21
3123.96	4235.71	5623.10	7164.86	8926.40	10856.50	13018.26	15648.51	19432.36
3127.52	4240.53	5629.76	7173.55	8937.05	10862.63	13039.38	15660.07	19444.31
3135.35	4247.52	5637.14	7181.66	8947.25	10871.97	13056.66	15668.17	19468.80
3140.92	4258.11	5643.47	7185.73	8958.28	10881.10	13070.53	15676.34	19499.63
3144.25	4263.40	5654.00	7189.92	8971.70	10893.40	13082.12	15690.07	19533.25
3148.87	4271.11	5661.05	7197.36	8985.80	10900.73	13100.47	15707.29	19558.66
3152.93	4277.34	5670.84	7210.71	8992.23	10909.10	13112.88	15717.91	19582.41
3155.84	4291.53	5679.37	7218.73	9000.38	10920.91	13124.12	15728.22	19612.80
3160.30	4299.89	5685.70	7230.96	9009.06	10929.81	13142.05	15743.57	19633.56
3164.95	4313.70	5696.71	7240.97	9014.82	10941.47	13157.38	15752.02	19661.08
3168.30	4323.42	5706.63	7246.69	9028.04	10952.26	13164.43	15761.73	19694.84
3173.40	4327.82	5715.98	7254.02	9039.40	10969.09	13182.26	15774.07	19730.91
3176.47	4335.41	5720.72	7262.69	9044.46	10977.85	13207.47	15789.95	19783.91
3181.05	4340.07	5725.00	7271.17	9058.62	10986.77	13229.65	15817.88	19824.99
3186.34	4348.31	5729.60	7281.29	9068.64	10997.08	13242.66	15840.21	19852.13
3202.04	4354.49	5733.74	7285.76	9086.74	11009.83	13255.55	15865.88	19879.41
3205.00	4358.26	5738.50	7289.97	9097.23	11019.98	13267.05	15881.52	19899.39
3210.49	4368.04	5745.86	7295.51	9106.56	11029.54	13283.27	15901.77	19919.82
3221.10	4372.18	5755.54	7308.21	9114.71	11034.99	13305.18	15921.70	19937.12
3227.04	4385.97	5761.75	7321.48	9133.96	11048.63	13327.33	15931.99	19951.82
3230.81	4392.93	5768.41	7330.08	9145.86	11058.52	13347.81	15940.22	19967.41
3243.68	4400.64	5772.44	7342.90	9153.34	11066.80	13369.86	15963.74	20018.90
3249.51	4405.68	5778.27	7355.61	9164.01	11077.83	13389.37	16000.78	20035.33
3253.77	4413.05	5786.51	7365.39	9171.95	11087.71	13404.29	16023.32	20085.41
3259.93	4419.43	5791.13	7373.42	9177.34	11093.24	13421.25	16043.80	20105.84
3264.92	4426.20	5807.89	7384.77	9183.79	11100.71	13433.97	16063.46	20164.10
3269.89	4440.72	5817.51	7393.66	9191.13	11114.05	13440.15	16077.67	20187.60
3273.90	4451.82	5821.38	7399.14	9197.97	11121.79	13464.36	16086.82	20211.92
3280.54	4464.93	5831.77	7408.13	9209.81	11130.73	13472.86	16105.74	20239.18
3289.41	4475.22	5837.29	7423.38	9215.86	11143.07	13483.03	16128.22	20262.40
3295.87	4481.01	5842.64	7433.78	9223.72	11149.00	13493.85	16142.93	20279.52
3300.58	4498.00	5850.22	7438.03	9232.63	11158.50	13502.96	16159.83	20300.93
3314.31	4506.19	5868.62	7451.00	9243.12	11175.95	13510.90	16176.29	20336.51

3320.18	4514.34	5873.54	7458.61	9251.98	11191.91	13518.56	16186.62	20373.85
3327.17	4521.24	5884.32	7462.56	9271.35	11204.14	13533.67	16207.26	20397.31
3332.07	4527.24	5890.06	7480.53	9293.26	11216.31	13550.31	16220.73	20412.22
3334.80	4533.62	5899.26	7487.18	9301.33	11228.33	13569.59	16249.42	20498.11
3340.24	4538.82	5904.97	7494.57	9305.77	11239.81	13577.13	16269.93	20533.28
3348.91	4542.85	5911.80	7499.22	9312.29	11251.38	13599.08	16291.41	20547.75
3355.69	4547.29	5917.46	7515.80	9327.09	11264.71	13620.85	16312.82	20577.94
3359.22	4554.42	5923.35	7522.54	9337.60	11271.48	13630.51	16331.52	20619.70
3362.86	4562.38	5927.55	7528.95	9349.53	11278.90	13642.53	16355.80	20640.39
3369.62	4569.72	5931.07	7538.39	9361.46	11290.74	13651.65	16385.56	20657.18
3374.79	4573.32	5939.32	7546.59	9371.31	11303.25	13667.87	16401.18	20697.20
3379.93	4577.91	5946.45	7551.57	9383.43	11316.16	13688.02	16433.17	20750.43
3383.41	4584.70	5952.60	7563.04	9390.16	11323.16	13697.53	16446.03	20800.19
3389.13	4594.27	5958.41	7573.98	9404.08	11334.27	13710.71	16455.11	20856.44
3401.96	4604.35	5962.92	7584.72	9420.97	11344.06	13732.19	16470.83	20877.48
3406.53	4609.34	5971.71	7595.21	9433.39	11349.35	13740.58	16494.83	20948.61
3411.71	4616.51	5976.28	7606.80	9439.36	11356.30	13757.78	16515.32	20970.63
3424.49	4620.87	5982.68	7614.72	9447.50	11361.13	13774.56	16532.23	21003.36
3433.18	4627.38	5987.67	7620.05	9458.62	11374.00	13789.98	16547.02	21053.71
3446.37	4633.99	5996.88	7626.05	9466.95	11383.69	13803.75	16556.90	21082.96
3452.02	4646.35	6010.71	7648.33	9476.39	11394.08	13821.44	16569.96	21111.63
3455.68	4651.75	6016.38	7654.27	9497.31	11402.94	13833.78	16585.81	21181.96
3469.03	4659.58	6025.41	7664.25	9507.58	11420.02	13852.73	16599.13	21208.46
3474.83	4663.98	6037.87	7677.39	9515.26	11431.42	13873.83	16617.50	21249.65
3484.65	4671.95	6043.26	7687.42	9523.74	11438.59	13885.27	16630.27	21272.12
3489.61	4678.05	6048.20	7696.05	9540.08	11447.53	13893.75	16642.09	21295.11
3495.35	4684.28	6055.09	7705.19	9548.92	11455.54	13912.00	16653.43	21336.49
3501.48	4692.60	6060.09	7718.72	9564.86	11469.54	13923.35	16666.54	21400.55
3511.92	4699.16	6067.66	7728.05	9569.48	11476.98	13934.42	16680.77	21429.32
3518.01	4703.41	6073.11	7734.54	9576.22	11487.26	13951.14	16691.55	21451.92
3522.70	4709.05	6077.70	7747.68	9583.71	11504.56	13981.14	16715.28	21478.44
3528.17	4717.81	6083.64	7756.63	9595.03	11513.72	13988.41	16734.54	21513.16
3532.84	4728.78	6091.87	7761.22	9607.13	11526.17	13995.82	16746.54	21545.01
3538.35	4734.02	6099.41	7768.27	9629.54	11540.16	14005.38	16763.81	21587.21
3544.20	4737.23	6108.95	7774.06	9639.70	11556.08	14038.58	16780.51	21621.32
3556.48	4740.13	6118.73	7780.23	9654.28	11564.31	14053.80	16806.21	21641.08
3564.40	4745.39	6124.62	7785.67	9666.21	11571.09	14069.99	16821.39	21724.78
3571.95	4762.64	6132.56	7792.91	9672.84	11582.81	14100.71	16831.43	21752.41
3577.28	4766.75	6139.41	7804.22	9680.52	11591.58	14117.91	16850.08	21776.02
3581.37	4770.83	6151.52	7809.06	9698.38	11605.17	14141.20	16865.97	21833.48
3584.85	4782.63	6160.34	7815.21	9709.91	11617.71	14158.81	16878.90	21872.68
3588.61	4792.74	6166.79	7823.26	9716.08	11629.50	14166.12	16897.69	21920.34
3593.43	4797.32	6170.71	7832.66	9724.29	11638.86	14177.63	16910.48	22003.32

3600.15	4803.10	6174.22	7841.43	9736.89	11660.46	14185.90	16921.92	22042.97
3606.98	4811.25	6183.51	7845.72	9747.47	11667.06	14192.75	16939.60	22106.28
3611.44	4820.31	6191.82	7855.08	9755.71	11681.18	14206.31	16955.56	22198.43
3615.71	4831.02	6196.06	7863.60	9767.41	11695.11	14216.57	16967.04	22235.32
3618.27	4843.68	6206.01	7874.07	9786.02	11708.36	14247.85	16977.90	22281.82
3622.43	4848.23	6216.14	7880.06	9798.25	11714.99	14261.15	16995.97	22314.00
3625.86	4851.57	6220.58	7888.38	9809.06	11722.08	14273.48	17016.50	22351.79
3630.28	4854.98	6224.70	7908.10	9825.22	11733.54	14281.44	17038.64	22392.03
3634.94	4862.99	6229.24	7922.06	9838.44	11741.91	14290.27	17059.51	22425.91
3639.55	4873.37	6234.77	7934.13	9845.87	11751.22	14304.25	17086.32	22467.06
3645.26	4883.23	6243.74	7944.64	9861.04	11759.76	14312.20	17105.15	22500.03
3650.77	4891.02	6249.64	7955.53	9872.00	11774.33	14322.89	17127.12	22522.30
3658.97	4900.11	6254.01	7965.72	9882.14	11789.53	14332.43	17138.94	22585.61
3663.05	4908.30	6263.55	7976.00	9888.43	11795.86	14342.40	17150.37	22620.46
3667.71	4919.84	6269.41	7987.12	9896.21	11801.93	14355.46	17165.22	22741.20
3672.17	4928.80	6280.15	8002.97	9905.28	11816.73	14366.84	17186.42	22814.15
3675.45	4936.47	6285.78	8017.49	9910.30	11827.50	14383.31	17202.33	22852.96
3683.97	4944.02	6295.73	8023.63	9922.58	11841.13	14395.17	17215.12	22909.25
3687.74	4951.26	6306.85	8035.39	9929.44	11859.60	14417.34	17267.65	22999.68
3694.31	4959.06	6316.35	8040.13	9937.54	11873.25	14432.82	17289.48	23045.33
3699.91	4962.66	6325.20	8047.41	9945.49	11881.10	14446.79	17305.99	23187.30
3703.88	4967.30	6339.42	8057.94	9956.08	11888.28	14459.91	17330.88	23223.56
3711.40	4974.73	6344.42	8068.41	9966.93	11904.65	14469.42	17344.88	23263.90
3718.14	4979.50	6354.03	8075.59	9981.71	11918.37	14482.76	17368.19	23288.42
3722.91	4984.44	6362.32	8088.95	9993.41	11932.13	14496.60	17395.72	23402.84
3728.02	4993.28	6368.13	8100.31	10001.96	11948.06	14511.55	17419.81	23437.59
3731.20	5001.91	6376.69	8107.70	10015.71	11963.27	14523.32	17430.14	23479.48
3734.58	5010.16	6380.79	8114.78	10032.39	11974.73	14533.21	17439.39	23507.03
3738.56	5016.93	6389.87	8122.07	10041.13	11984.40	14547.36	17449.07	23551.79
3745.33	5028.55	6393.71	8128.89	10046.84	11995.85	14560.02	17472.94	23588.38
3749.35	5038.49	6400.45	8134.07	10057.42	12006.04	14567.02	17491.05	23612.33
3760.93	5045.11	6408.83	8139.59	10067.98	12013.33	14578.65	17503.68	23646.04
3766.25	5051.35	6432.36	8149.23	10084.83	12021.43	14605.09	17558.57	23680.48
3771.15	5058.30	6442.41	8158.65	10092.68	12037.17	14616.22	17571.10	23707.52
3782.31	5064.91	6451.04	8168.83	10100.64	12051.39	14622.87	17590.51	23812.66
3787.19	5070.17	6456.16	8179.37	10108.92	12062.54	14630.13	17601.88	23843.75
3791.47	5073.91	6464.17	8196.52	10116.78	12075.16	14640.85	17623.48	23878.89
3794.82	5081.99	6473.00	8204.89	10122.48	12082.12	14654.25	17636.21	23932.17
3799.71	5091.02	6482.59	8215.21	10128.31	12095.33	14680.10	17657.85	24012.76
3805.50	5101.30	6486.58	8234.59	10136.50	12102.60	14696.56	17677.86	24042.28
3809.31	5108.54	6497.42	8243.23	10145.66	12109.05	14705.46	17696.03	24093.05
3814.85	5115.79	6507.49	8254.56	10152.02	12122.94	14715.29	17776.40	24180.24
3817.90	5122.77	6512.82	8261.31	10157.05	12131.44	14727.41	17793.87	24233.88

3823.15	5128.76	6529.74	8267.61	10165.16	12138.54	14738.49	17813.85	24267.91
3831.32	5138.60	6541.28	8280.04	10170.63	12151.34	14747.95	17828.16	24305.19
3838.30	5144.71	6551.13	8288.77	10178.17	12167.42	14755.05	17842.48	24422.87
3843.61	5152.03	6565.33	8296.19	10184.68	12178.18	14774.79	17861.04	24590.98
3848.40	5163.00	6574.97	8308.54	10196.80	12183.55	14792.36	17883.74	24658.45
3856.77	5166.94	6581.20	8316.06	10208.87	12191.83	14809.58	17902.08	24886.03
3860.22	5177.26	6597.23	8323.67	10218.15	12207.49	14819.58	17925.32	24925.21
3866.70	5182.27	6605.37	8337.55	10224.94	12217.27	14831.54	17957.30	24956.47
3873.56	5187.95	6624.95	8351.27	10230.63	12238.46	14842.77	17977.54	25200.43
3880.02	5192.56	6632.02	8359.97	10236.29	12252.80	14853.53	18001.11	25371.89
3882.83	5197.58	6641.07	8370.76	10242.72	12262.81	14865.60	18011.46	25409.10
3886.86	5203.82	6663.74	8383.62	10264.83	12271.10	14873.23	18027.80	25487.72
3891.80	5212.88	6678.21	8396.33	10270.33	12280.64	14900.61	18051.11	25544.81
3898.81	5218.43	6684.38	8409.78	10278.82	12293.22	14909.44	18085.22	25583.42
3912.38	5227.64	6700.67	8419.09	10290.93	12300.45	14918.81	18108.20	25639.25
3917.40	5234.23	6706.53	8428.16	10312.44	12307.71	14928.74	18121.09	25699.96
3922.47	5238.03	6711.86	8437.12	10321.54	12327.25	14941.84	18161.76	25838.88
3928.93	5246.53	6728.38	8450.42	10328.72	12343.08	14951.25	18183.02	25892.55
3938.06	5254.38	6738.89	8457.33	10345.42	12350.79	14968.25	18204.64	25926.90
3941.32	5261.22	6751.08	8462.53	10352.79	12361.39	14979.26	18234.10	25951.15
3947.65	5271.22	6755.68	8474.21	10361.39	12376.42	14999.38	18259.18	25976.35
3954.51	5275.66	6770.11	8485.50	10374.58	12395.82	15017.08	18283.36	26006.76
3960.83	5280.52	6783.86	8496.04	10385.78	12409.90	15028.40	18311.33	26147.35
3964.82	5284.51	6794.91	8512.50	10391.00	12422.97	15038.32	18333.95	26325.93
3968.25	5290.58	6802.85	8520.10	10396.86	12443.01	15057.95	18356.20	26541.84
3971.85	5298.93	6808.68	8532.29	10408.56	12456.12	15069.63	18380.08	26664.91
3977.72	5305.56	6814.79	8538.37	10426.05	12462.38	15080.51	18393.89	26755.99
3984.29	5310.27	6820.73	8551.92	10434.61	12480.81	15092.78	18411.80	26804.25
3989.90	5318.39	6827.49	8556.91	10445.30	12494.74	15105.40	18459.82	26838.68
3995.35	5323.49	6840.96	8561.14	10458.20	12502.78	15124.65	18477.65	26864.30
4004.94	5328.07	6847.23	8565.68	10467.56	12517.60	15140.54	18504.82	26948.97
4010.12	5336.49	6858.73	8574.02	10476.61	12529.33	15159.16	18530.51	27000.27
4013.43	5341.15	6868.82	8586.89	10489.25	12539.89	15166.98	18591.76	27114.14
4017.19	5347.26	6881.00	8595.72	10501.86	12557.04	15180.97	18615.71	27519.82
4023.49	5356.15	6890.98	8603.11	10517.47	12568.58	15201.03	18630.97	27577.14
4028.91	5369.87	6902.61	8613.58	10534.69	12585.17	15229.15	18644.61	27700.21
4038.68	5376.44	6910.24	8619.60	10544.70	12603.68	15240.03	18669.62	27972.94
4048.04	5385.89	6923.19	8637.49	10560.85	12620.97	15250.33	18690.96	28101.79
4057.13	5393.72	6930.78	8647.96	10569.52	12634.02	15260.63	18708.36	28174.21
4062.29	5401.18	6941.36	8654.70	10575.74	12644.12	15270.98	18723.27	28304.66
4076.49	5415.07	6950.64	8667.38	10590.94	12667.15	15286.87	18742.14	28512.26
4080.34	5424.55	6964.98	8681.90	10605.56	12683.81	15302.64	18774.44	28596.77
4084.60	5437.86	6971.27	8693.25	10614.61	12702.05	15321.46	18790.47	28732.48

4090.59	5447.04	6978.90	8702.19	10623.47	12707.58	15338.57	18813.57	28891.36
4097.59	5458.29	6987.20	8712.55	10632.72	12719.39	15345.95	18838.92	28976.55
4105.77	5463.20	6993.54	8724.28	10647.90	12733.63	15364.12	18857.45	29089.78
4114.17	5467.72	7003.17	8730.42	10657.15	12745.35	15377.10	18874.15	29181.73
4116.91	5477.53	7008.25	8737.38	10673.58	12757.69	15385.38	18900.85	29665.27
4121.23								

Table S8 – Peak list for SimulTOF100

Table S8. Peak list for the SimulTOF100 consisting of 1256 peaks.

3023.81	5250.33	6968.65	8487.70	10118.77	11478.13	13205.15	15394.02	18441.89
3044.41	5267.27	6985.57	8500.67	10125.23	11484.01	13216.19	15409.92	18474.73
3063.74	5275.15	6993.09	8513.06	10130.20	11498.14	13231.48	15418.39	18496.65
3073.50	5291.35	6999.82	8520.93	10137.19	11506.46	13245.58	15436.45	18555.68
3088.91	5308.43	7022.62	8528.39	10147.56	11513.70	13259.62	15452.03	18572.75
3111.73	5316.85	7033.88	8545.37	10155.58	11518.63	13273.35	15471.85	18598.67
3142.77	5321.99	7044.49	8554.72	10172.58	11530.28	13291.50	15485.24	18611.75
3153.94	5327.21	7055.35	8563.30	10181.39	11540.65	13297.45	15501.05	18629.85
3168.21	5336.33	7065.31	8571.97	10191.16	11550.19	13315.73	15513.73	18654.26
3179.35	5344.02	7072.08	8581.23	10198.24	11566.50	13335.09	15530.66	18708.90
3202.34	5362.27	7084.18	8592.31	10208.31	11572.34	13344.50	15539.63	18725.60
3218.78	5375.13	7091.47	8615.38	10213.01	11582.69	13358.54	15558.76	18756.72
3242.75	5381.06	7096.63	8626.71	10218.96	11591.43	13372.47	15576.48	18796.92
3266.26	5393.90	7101.40	8637.82	10225.77	11600.23	13388.78	15604.42	18839.78
3292.17	5407.51	7127.20	8657.07	10232.79	11606.76	13399.57	15627.53	18858.47
3318.13	5419.30	7137.82	8682.70	10244.73	11628.16	13410.57	15646.20	18893.00
3334.86	5430.24	7146.62	8691.85	10251.86	11651.76	13429.28	15659.78	19021.89
3367.23	5452.15	7151.34	8701.89	10259.99	11676.53	13455.28	15675.28	19039.04
3398.13	5461.05	7156.71	8712.10	10271.34	11682.58	13469.94	15692.63	19054.77
3422.99	5473.53	7164.76	8724.31	10276.39	11697.46	13479.24	15709.04	19079.28
3431.74	5493.60	7176.07	8739.63	10283.00	11705.01	13486.42	15719.44	19093.95
3446.26	5504.62	7187.51	8752.36	10291.99	11712.67	13498.16	15732.55	19142.31
3467.03	5511.98	7202.06	8765.88	10300.52	11721.33	13507.65	15744.69	19240.76
3484.77	5522.54	7215.80	8785.63	10308.93	11730.60	13525.78	15758.79	19278.06
3497.59	5536.06	7244.05	8791.08	10317.96	11741.68	13538.28	15776.37	19310.09
3510.51	5556.81	7251.64	8796.47	10323.93	11750.28	13559.11	15869.80	19377.97
3555.38	5574.29	7259.34	8801.53	10334.34	11763.43	13568.18	15890.70	19435.37
3582.41	5585.97	7270.68	8810.93	10342.10	11783.63	13578.13	15915.82	19451.06
3592.95	5600.64	7287.77	8822.01	10352.73	11794.07	13588.06	15947.19	19469.51
3612.67	5614.99	7299.24	8826.50	10360.98	11808.84	13595.30	15967.18	19502.69
3645.54	5619.75	7306.78	8838.84	10371.92	11829.33	13608.80	15987.99	19562.55
3665.97	5630.55	7311.88	8848.06	10378.07	11839.10	13628.21	16010.26	19593.10
3680.98	5635.81	7318.38	8859.27	10384.35	11846.96	13637.38	16033.45	19672.00

3692.81	5643.88	7331.83	8868.42	10391.37	11861.84	13651.74	16054.40	19725.43
3705.82	5657.78	7343.94	8891.39	10401.90	11871.63	13660.34	16075.80	19776.10
3725.12	5663.26	7356.70	8915.96	10411.25	11877.31	13678.51	16094.11	19814.18
3737.29	5677.53	7375.66	8927.45	10421.56	11891.01	13687.35	16114.60	19833.29
3757.69	5685.81	7389.02	8933.91	10429.52	11906.56	13700.55	16132.75	19851.06
3778.03	5697.80	7398.48	8946.68	10437.33	11919.14	13723.73	16150.94	19891.29
3804.20	5708.35	7416.92	8953.09	10445.98	11938.34	13747.90	16176.02	19911.28
3811.16	5719.95	7425.32	8959.01	10458.00	11952.76	13762.96	16197.52	19933.45
3823.73	5735.65	7437.91	8973.21	10468.46	11968.34	13778.07	16217.31	19975.88
3831.71	5751.55	7451.76	8980.82	10477.04	11980.30	13792.38	16236.56	19989.33
3843.01	5764.10	7460.58	8995.80	10491.07	11988.72	13804.35	16259.24	20033.11
3867.87	5778.03	7469.80	9017.64	10505.77	11998.19	13810.37	16281.63	20071.40
3875.27	5794.17	7477.57	9026.20	10515.81	12005.62	13824.12	16301.17	20162.91
3892.40	5801.44	7483.87	9034.43	10525.08	12010.72	13842.89	16317.77	20197.18
3908.68	5812.68	7488.43	9048.33	10532.48	12026.62	13869.14	16339.78	20324.42
3923.71	5823.15	7497.21	9062.17	10546.72	12039.90	13881.83	16347.99	20356.18
3935.75	5841.90	7503.36	9070.94	10557.81	12050.87	13899.99	16368.99	20395.18
3955.82	5853.08	7508.49	9076.79	10567.31	12062.40	13911.55	16392.04	20513.05
3967.68	5864.03	7514.83	9084.76	10581.70	12072.56	13926.61	16434.61	20538.45
3979.17	5878.19	7521.00	9095.34	10593.12	12083.79	13938.62	16457.01	20576.65
3989.64	5884.16	7536.45	9107.13	10600.05	12089.10	13977.14	16483.32	20603.92
4012.99	5890.12	7553.18	9123.98	10607.49	12094.84	13993.57	16504.56	20634.89
4033.40	5896.37	7557.55	9135.14	10613.50	12107.07	14029.31	16523.52	20654.67
4052.15	5909.74	7563.70	9152.93	10623.28	12114.95	14041.72	16534.38	20767.09
4077.30	5920.83	7573.08	9165.39	10632.85	12120.80	14057.76	16556.93	20794.42
4088.90	5928.35	7584.92	9177.19	10646.35	12129.80	14069.96	16634.10	20835.26
4102.00	5937.29	7596.16	9183.31	10663.77	12138.62	14090.21	16651.92	20927.52
4122.29	5955.41	7607.01	9192.45	10675.42	12148.81	14106.43	16661.03	20957.98
4134.49	5965.89	7615.43	9203.65	10680.53	12154.84	14117.41	16672.56	20974.81
4154.25	5988.52	7628.66	9213.27	10688.13	12161.22	14138.70	16692.64	21039.39
4173.58	6007.68	7647.64	9222.12	10695.45	12167.40	14149.21	16711.05	21065.63
4186.52	6028.37	7653.43	9241.33	10701.36	12174.22	14167.71	16736.89	21107.57
4197.23	6046.90	7666.92	9261.77	10708.43	12180.73	14194.97	16775.90	21165.46
4209.59	6064.12	7676.86	9279.37	10726.70	12196.82	14218.34	16795.38	21267.61
4216.53	6077.38	7690.10	9287.56	10735.10	12205.97	14229.76	16814.61	21368.85
4230.56	6088.79	7696.38	9293.67	10745.46	12212.52	14254.19	16824.85	21400.32
4240.08	6095.40	7709.25	9302.78	10751.67	12230.53	14274.02	16837.37	21451.11
4251.74	6109.57	7715.29	9316.84	10771.77	12238.42	14296.08	16868.42	21474.89
4265.68	6122.72	7721.24	9325.99	10780.79	12248.43	14308.21	16885.71	21570.97
4289.97	6141.99	7737.52	9352.49	10789.39	12258.30	14327.58	16898.19	21609.06
4307.97	6148.05	7747.51	9361.52	10795.98	12273.83	14342.34	16916.81	21705.78
4316.15	6153.82	7758.20	9373.47	10801.49	12280.73	14355.99	16929.40	21756.33
4342.58	6174.21	7768.73	9387.98	10811.00	12295.00	14369.82	16948.31	21803.77

4355.45	6187.31	7781.66	9394.49	10829.65	12312.88	14379.64	16963.03	21852.46
4361.71	6196.51	7799.61	9412.24	10836.72	12340.76	14403.22	16984.62	21909.01
4383.46	6212.04	7811.59	9422.82	10851.96	12350.86	14421.73	16998.35	21980.40
4391.94	6225.90	7822.21	9427.55	10872.41	12366.32	14437.88	17024.39	22024.60
4406.18	6233.39	7829.63	9436.04	10878.95	12374.45	14451.55	17038.98	22084.70
4418.37	6244.98	7839.84	9445.47	10887.38	12383.24	14462.48	17050.93	22189.95
4435.49	6254.24	7847.39	9453.56	10902.79	12391.96	14473.04	17066.47	22223.59
4442.80	6275.41	7852.88	9462.29	10912.64	12403.83	14485.29	17086.12	22260.52
4458.66	6286.77	7867.73	9480.96	10920.45	12411.08	14498.74	17105.70	22296.83
4470.23	6299.03	7877.91	9487.18	10929.39	12435.84	14512.64	17125.82	22323.48
4487.22	6310.83	7897.96	9501.31	10934.29	12454.26	14529.37	17151.93	22351.04
4508.97	6317.44	7912.71	9507.70	10944.16	12467.06	14545.23	17175.38	22388.85
4521.16	6332.29	7934.18	9512.41	10956.44	12485.23	14563.09	17198.37	22425.11
4537.23	6344.28	7944.90	9529.85	10963.16	12491.63	14570.34	17226.58	22465.33
4547.71	6350.41	7955.32	9538.34	10968.06	12503.31	14583.17	17253.95	22498.41
4566.83	6356.97	7959.88	9557.64	10979.12	12513.03	14598.41	17277.10	22529.32
4586.66	6369.46	7965.16	9573.85	10991.44	12519.54	14630.28	17310.57	22550.44
4598.41	6389.13	7979.90	9585.83	11000.24	12542.40	14641.85	17332.61	22605.63
4613.77	6399.36	7992.88	9597.12	11007.63	12558.09	14656.02	17360.43	22707.75
4627.63	6421.40	7997.83	9623.28	11017.02	12567.55	14668.79	17381.97	22798.92
4644.35	6433.04	8015.52	9629.58	11026.18	12573.82	14693.07	17407.88	22830.64
4666.75	6443.52	8025.91	9645.64	11044.35	12589.22	14704.19	17434.97	22909.04
4676.43	6453.70	8037.42	9660.09	11059.40	12608.21	14717.37	17460.77	22967.33
4688.85	6472.68	8047.12	9668.82	11066.08	12624.71	14728.57	17479.35	23030.92
4698.77	6481.77	8058.26	9687.41	11070.83	12635.10	14738.85	17491.04	23134.10
4711.59	6494.28	8065.84	9701.70	11078.51	12641.03	14751.93	17548.46	23169.62
4723.37	6516.78	8075.29	9714.05	11088.24	12656.84	14767.25	17590.01	23250.10
4737.61	6531.52	8088.40	9727.56	11095.21	12672.27	14780.35	17610.37	23316.23
4756.33	6540.92	8103.36	9736.84	11101.41	12689.91	14794.69	17626.71	23354.17
4776.68	6556.30	8119.65	9746.34	11107.68	12707.30	14808.63	17643.24	23463.84
4790.70	6565.17	8128.13	9757.68	11125.98	12734.02	14818.77	17654.27	23557.18
4815.49	6587.09	8139.94	9775.66	11137.61	12744.68	14832.17	17669.91	23667.26
4823.51	6595.25	8149.69	9789.57	11147.86	12754.30	14845.03	17682.14	23805.39
4831.57	6623.54	8159.04	9797.66	11158.48	12769.17	14860.15	17699.40	23846.17
4839.80	6631.39	8167.35	9806.36	11163.59	12780.24	14878.48	17729.30	24118.20
4856.73	6641.41	8172.01	9816.46	11169.71	12795.60	14898.96	17751.99	24645.21
4870.38	6652.13	8187.24	9834.47	11176.06	12849.06	14914.02	17772.56	25185.27
4880.37	6666.07	8191.97	9852.36	11188.02	12857.30	14931.67	17792.59	25274.04
4892.61	6671.88	8204.54	9861.39	11193.01	12865.01	14940.63	17804.16	25402.98
4910.97	6682.75	8216.91	9870.51	11204.25	12879.06	14953.82	17827.45	25477.33
4923.42	6687.77	8222.73	9879.33	11217.05	12891.28	14972.04	17844.64	25558.75
4938.12	6693.68	8230.12	9891.22	11226.74	12905.53	14987.38	17873.99	25613.20
4961.54	6720.43	8237.02	9904.39	11233.81	12914.53	15005.88	17891.36	25674.54

4973.11	6729.54	8244.58	9919.34	11245.41	12923.75	15020.22	17911.45	25800.92
4979.80	6739.39	8257.66	9935.11	11255.65	12933.20	15038.71	17960.03	25898.64
4997.37	6751.41	8268.51	9949.88	11263.86	12948.66	15045.48	17983.85	25947.39
5023.73	6757.85	8279.50	9955.27	11277.47	12963.47	15053.87	18016.28	26861.45
5034.83	6774.27	8285.58	9963.58	11289.56	12979.10	15074.85	18040.00	27406.52
5044.88	6785.06	8294.01	9969.79	11302.92	12991.38	15128.49	18061.88	27564.84
5054.96	6794.61	8304.16	9981.61	11312.67	13007.11	15146.32	18080.85	27965.03
5068.88	6806.88	8314.08	9992.01	11317.28	13030.24	15165.79	18098.57	28094.69
5077.76	6815.14	8327.31	10005.54	11339.94	13046.65	15180.43	18126.82	28191.16
5095.59	6837.92	8334.99	10011.56	11352.60	13060.40	15207.42	18145.45	28299.46
5107.54	6858.87	8340.67	10018.75	11362.04	13069.36	15217.48	18170.40	28402.00
5117.48	6871.48	8346.18	10024.30	11370.72	13088.39	15227.33	18191.80	28511.56
5129.09	6881.02	8360.44	10034.00	11379.83	13104.12	15247.13	18211.37	28611.98
5142.83	6895.92	8370.26	10049.13	11388.81	13109.62	15277.94	18238.77	28716.59
5151.64	6903.33	8388.44	10058.47	11395.76	13115.26	15292.68	18269.58	28879.68
5157.18	6909.16	8410.51	10064.03	11408.05	13130.02	15313.23	18290.27	28971.42
5176.91	6921.52	8427.55	10071.50	11434.22	13154.57	15325.12	18338.74	29082.43
5195.87	6928.95	8442.58	10079.97	11443.30	13168.89	15334.40	18369.56	29188.23
5206.08	6940.54	8448.43	10085.15	11450.69	13178.52	15352.17	18389.18	29287.76
5223.54	6954.18	8462.93	10099.13	11458.08	13188.85	15371.02	18418.11	29656.50
5231.12	6961.15	8474.97	10110.35	11466.52				

SI References

1. Senko, M.W.; Beu, S.C.; McLaffertycor, F.W. Determination of Monoisotopic Masses and Ion Populations for Large Biomolecules from Resolved Isotopic Distributions. *J. Am. Soc. Mass Spectrom.* **1995**, *6*, 229–233, doi:10.1016/1044-0305(95)00017-8.
